# Recent advances of fluorescent biosensors based on cyclic signal amplification technology in biomedical detection

**DOI:** 10.1186/s12951-021-01149-z

**Published:** 2021-12-04

**Authors:** Hongke Qu, Chunmei Fan, Mingjian Chen, Xiangyan Zhang, Qijia Yan, Yumin Wang, Shanshan Zhang, Zhaojian Gong, Lei Shi, Xiayu Li, Qianjin Liao, Bo Xiang, Ming Zhou, Can Guo, Guiyuan Li, Zhaoyang Zeng, Xu Wu, Wei Xiong

**Affiliations:** 1 NHC Key Laboratory of Carcinogenesis and Hunan Key Laboratory of Cancer Metabolism, Hunan Cancer Hospital and The Affiliated Cancer Hospital of Xiangya School of Medicine, Central South University, Changsha, Hunan China; 2 Key Laboratory of Carcinogenesis and Cancer Invasion of the Chinese Ministry of Education, Cancer Research Institute and School of Basic Medicine Sciences, Central South University, Changsha, Hunan China; 3 Hunan Key Laboratory of Nonresolving Inflammation and Cancer, Disease Genome Research Center, The Third Xiangya Hospital, Central South University, Changsha, Hunan China; 4 Department of Forensic Science, School of Basic Medical Sciences, Central South University, Changsha, Hunan China; 5 Department of Stomatology, Xiangya Hospital, Central South University, Changsha, Hunan China; 6 Department of Oral and Maxillofacial Surgery, The Second Xiangya Hospital, Central South University, Changsha, Hunan China

**Keywords:** Fluorescence biosensor, Cyclic signal amplification, Biomedical detection

## Abstract

The cyclic signal amplification technology has been widely applied for the ultrasensitive detection of many important biomolecules, such as nucleic acids, proteins, enzymes, adenosine triphosphate (ATP), metal ions, exosome, etc. Due to their low content in the complex biological samples, traditional detection methods are insufficient to satisfy the requirements for monitoring those biomolecules. Therefore, effective and sensitive biosensors based on cyclic signal amplification technology are of great significance for the quick and simple diagnosis and treatment of diseases. Fluorescent biosensor based on cyclic signal amplification technology has become a research hotspot due to its simple operation, low cost, short time, high sensitivity and high specificity. This paper introduces several cyclic amplification methods, such as rolling circle amplification (RCA), strand displacement reactions (SDR) and enzyme-assisted amplification (EAA), and summarizes the research progress of using this technology in the detection of different biomolecules in recent years, in order to provide help for the research of more efficient and sensitive detection methods.

**Supplementary Information:**

The online version contains supplementary material available at 10.1186/s12951-021-01149-z.

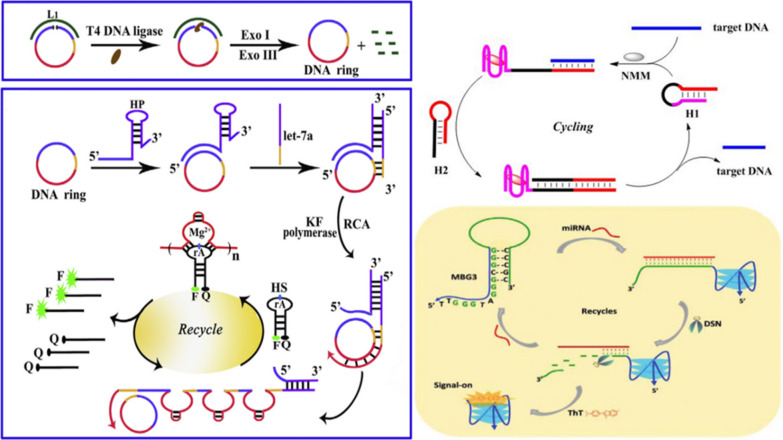

## Introduction

In the last decades, fluorescent biosensors have made great progress, and are widely used in biology, medicine, chemistry and other fields [1–5]. Biosensors based on fluorescence have many advantages, such as simple operation, fast response, simple instrument, multiple analysis, high sensitivity, and good selectivity [6–10]. In most fluorescent biosensors, the fluorescence intensity is recorded as the readout signal for the analytes. In order to obtain better performance of the fluorescent biosensors, several parameters, including the analyte recognition units, signal transducers, and fluorescent tags should be considered carefully. To improve the fluorescence signals of biosensors, a brighter fluorescent tag and signal amplification technology are usually applied during the construction.

For accurately analyzing trace biomolecules in biological samples, researchers explored the signal amplification strategies during the construction of fluorescent biosensors, which significantly enhanced the fluorescence signal and sensitivity of the biosensors [11]. Among the signal amplification strategies, cyclic signal amplification (CSA) technology is one of the most useful strategies due to its easy operation, low cost, and versatile design principles. CSA technology utilized the hybridization of nucleic acids or enzymes for nucleic acids to release the analyte, which will be recycled for further increase the fluorescence outputs of the biosensors. Therefore, after the CSA, the fluorescence signal could be amplified many folds to achieve highly sensitive and low limit of detection for biomolecules. The commonly used CSA methods include rolling circle amplification (RCA), strand displacement reactions (SDR) and enzyme-assisted amplification (EAA), etc. [12–14]. In continuous practice, researchers have also tried to combine multiple CSA methods together as well as simultaneous detection of multiple analytes to achieve better analytical performance for biomedical applications [15, 16].

With the development of fluorescent biosensors, a number of reviews focused on the recognition units and the fluorescent tags have been published in the last decade. For example, Yang et al. used aptamers to link gold nanoparticles and silver nanoclusters, and constructed a surface-enhanced fluorescence (SEF) strategy based on these two nanomaterials for the detection of carcinoembryonic antigen (CEA) with a detection limit of 3 pg mL^−1^ [17]. Xu et al. constructed the sensitive detection of mercury (II) based on the fluorescence sensing strategy of "molecular beacon" with 5-terminal labeling of 6-carboxyuorescein dye (FAM) and 3-terminal labeling of a quencher 4-(4-dimethylaminophenylazo) benzoic acid (DABCYL), and the detection limit was 2.5 nM [18]. However, the review emphasizing on the signal amplification strategies for fluorescent biosensor are rarely reported. Thus, in this review, we firstly summarize several CSA strategies, including rolling circle amplification, strand displacement reactions and enzyme-assisted amplification, etc. Moreover, we discuss the applications of the CSA-based fluorescent biosensors for different types of biomolecules. Finally, we discuss the major challenge and perspective of the fluorescence biosensors based on CSA strategies. We hope this review can provide a comprehensive view and inspire new research directions for the fabrication of sensitive fluorescent biosensor based on CSA strategies.

## Cyclic signal amplification strategies

In order to enhance the sensitivity and detection limit of fluorescent biosensors, more and more attention has been paid to the utilization of CSA technology during fabricating those biosensors. CSA technology can be roughly divided into three major categories according to the mechanism for amplification: rolling circle amplification, strand displacement reactions and enzyme-assisted amplification. The different types of cyclic signal amplification strategies and their properties are summarized in Table 1. In this section, we will discuss these different types of CSA strategies for fluorescent biosensors.

**Table 1:** Cyclic signal amplification strategies for fluorescent biosensors

Method	Characteristic	Advantages	Disadvantages	Real samples	References
RCA	Template, Primer, polymerization, amplification, release target	Good amplification effect, single reaction conditions, full use of reaction materials,	Long reaction times, requiring specific amplification template	Cells, serum, tissue, plasma, fecal, beer, drinking water	[23–25]
SDR	Hybridization, strand displacement, release target	No enzyme or cofactor, relatively simple experimental conditions, less external interference conditions, economical and cost-effective	Multiple probes or auxiliary strands need to be designed	Cells, serum, urine, milk, river water	[32, 33]
EAA	Hybridization, digestion, release target	No complex instrument, the signal is effective, the reaction is relatively thorough, and the time is short	Harsh reaction conditions, high temperature requirements, high cost of enzymes	Cells, serum, milk, artificial urine, river water	[35, 36]

### Rolling circle amplification

**Fig. 1:**
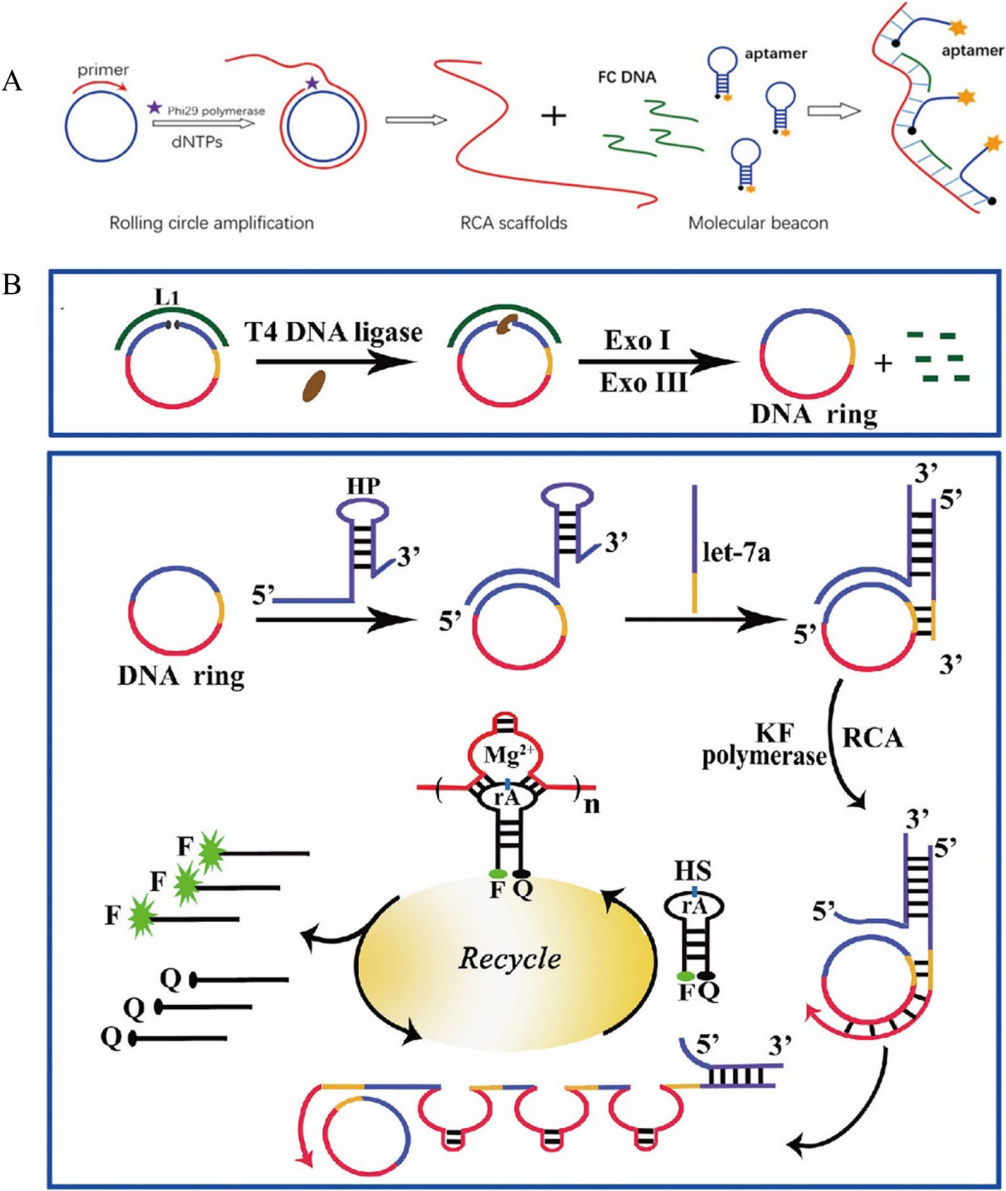
**A** Schematic illustration of circDNA as a template for RCA [21]. **B** Schematic diagram of sensitive detection of miRNA based on RCA (Linear DNA as a template) combined with DNAzyme [22]

As a relatively simple and powerful means of isothermal DNA replication, rolling circle amplification (RCA) is a commonly used signal amplification technology for constructing fluorescent biosensors [19, 20]. As shown in Fig. 1A, RCA contains a circular DNA and a linear complementary primer [21]. During the RCA process, circular DNA is used as a template and the primer is hybridized with the circular DNA, which then forms a single strand of DNA containing a large number of repeated sequences by polymerization, which can trigger the enhancement of the fluorescence signal through the amplification. The key elements of the RCA are the sequences of circular DNA and primer. Besides the circular DNA, long linear DNA can also be used as a template for RCA in the presence of DNA ligase. For example, Liu et al. demonstrated that a circular DNA could be formed in the presence of the ligase and the DNA (Fig. 1B) [22]. Then, with the addition of the prime DNA, RCA process was executed to generate numerous repeated DNA sequences. Furthermore, the DNA was released during this RCA process and could trigger another RCA process. At present, the circular template can be circular DNA or RNA. The research has shown that the RCA can amplify the fluorescence signal up to 1000–10,000 times, greatly improving the detection sensitivity [23–25].

Taking advantage of the isothermal amplification ability of RCA, many reports have been published for fabricating fluorescent biosensors based on RCA. For example, Liu et al*.* designed a hairpin/DNA ring ternary probe for highly selective and sensitive detection of microRNA (miRNA) by combining RCA with metal ion dependent DNA enzyme (DNAzyme) for signal amplification [22] (Fig. 1B). First, they used T4 DNA ligase, exonuclease I (Exo I) and exonuclease III (Exo III) to generate the circular DNA for the following RCA process. Then, the hairpin/DNA ring ternary probe was formed through hybridization reaction to form the probe responsive the miRNA. In the presence of the miRNA, RCA process was initiated with the addition of Klenow fragment enzyme and deoxynucleotide triphosphates (dNTPs). As a result of the RCA process, the DNA single strand containing a large number of repeated DNA sequences was obtained. With the assistance of Mg^2+^, DNAzyme cleaved the hairpin substrate (HS) probes labeled with both fluorophore and quencher, resulting in the recovery of fluorescence signal and release of the DNAzyme sequence, which reacted with the new HS probe to realize the purpose of cyclic amplification of fluorescent signal. With this amplification system, a highly sensitive detection of the miRNA was achieved, with the detection lower limit as low as 1.51 fM. This method can be used for measuring the changes of miRNA expression in cancer cells and selecting potential therapeutic drugs.

In addition, Gao et al*.* also constructed a sensitive fluorescent biosensor for the detection of cancer-related miRNA Let-7a based on the RCA strategy with the limit of detection at 10 pM [26]. Moreover, they showed that sensor could be used to detect miRNA Let-7a in the total RNA extracted from cells, which showed a highly expressed in Hela cells and a small amount in A549 cells. This method also has good reproducibility in fetal bovine serum, the relative standard deviation (RSD) was less than 9% and the recoveries were from 102 to 106% for different concentrations of let-7a. Recently, Jiang et al. developed an RCA-based sensor for the detection of miRNA using G-quadruplex structure combined with Thioflavin T (ThT) [27]. In the presence of the miRNA, a large G-quadruplex structure was generated through RCA, which was combined with THT to enhance fluorescence signal. The limit of detection for the miRNA with this fluorescent sensor was as low as 4 aM. This method can also be used for the detection of let-7a in human lung cancer cells (A549). Moreover, this method utilized a label-free probe, which reduced the cost and synthesis complexity compared with the labeled required sensors.

With the RCA process, a large number of repeated fragments will be generated by low copies of analytes. Moreover, the polymerase reaction might be able to release the analytes sequence during the RCA process, which can trigger another RCA process. The reaction conditions are relatively simple and easy to control with excellent amplification factor. It can make full use of reaction materials, and can greatly amplify the fluorescence signal in fluorescence detection. However, this technique usually takes a relative long reaction time and requires specific amplification templates [23–25, 28].

### Enzyme-free amplification strategies.

**Fig. 2:**
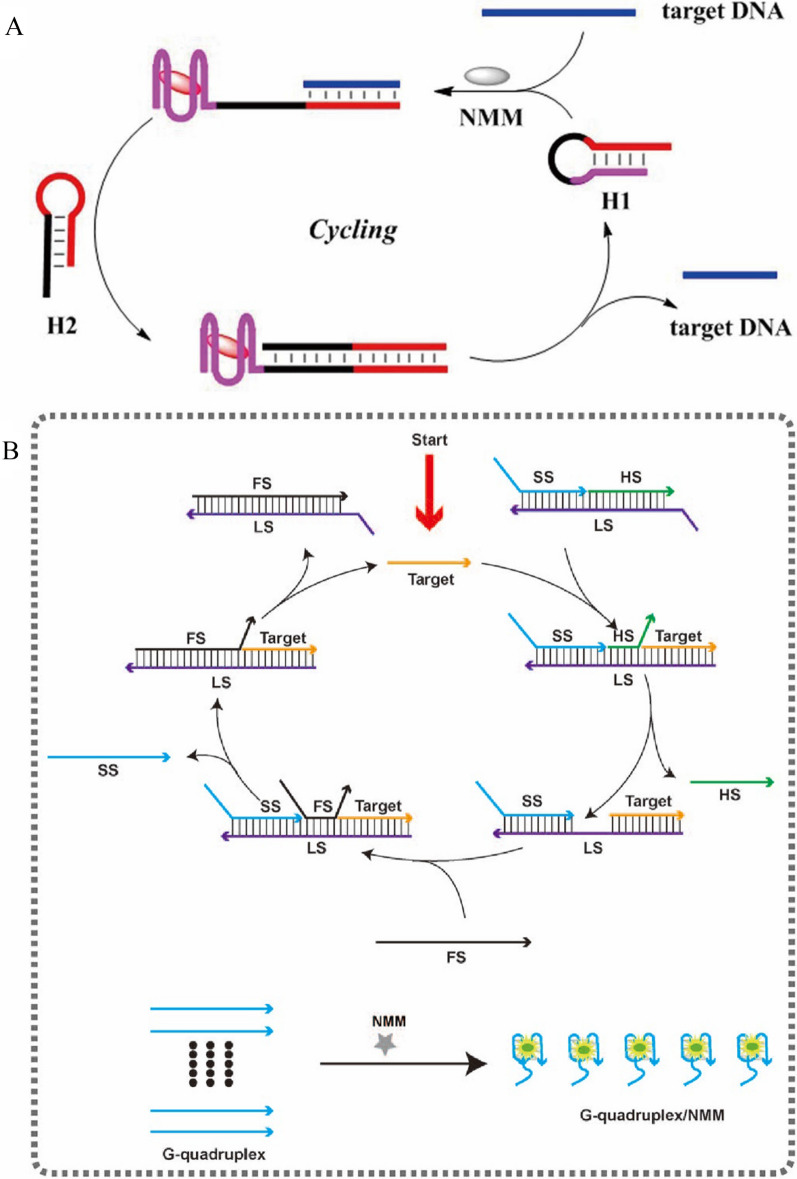
**A** The basic schematic diagram of enzyme-free strategy [29]. **B** Schematic of sensitive detection of HIV-1 DNA based on toehold-mediated SDR [30]

The strand displacement reaction (SDR) is an enzyme-free and co-factor free cyclic signal amplification, which works primarily through hybridization dynamics. SDR attracts more attention in biomolecules detection because it is cost-effective and less interference from external conditions, such as temperature, pH, and metal ions, etc. The basic reaction principle of SDR is that in the presence of the analyte, the analyte can hybridize with the probe to form an incomplete double-stranded structure, another probe or helper chain can then hybridize with the unhybridized portion of the original probe to release the analyte, and then the analyte can hybridize with the new probe to form a cycle process, resulting in a continuous enhancement of the fluorescent signal (Fig. 2A) [29].

As shown in Fig. 2B, Li et al. designed a cascading toehold-mediated SDR for sensitive detection of HIV-1 DNA [30]. The sensor contained a fuel strand (FS), a DNA substrate probe (SP), and N-methylmesoporphyrin IX (NMM). The SP was constructed by three DNA strands, which included a G-quadruplex signal strand (SS), helper strand (HL) and a long strand (LS). With the initiation by the presence of DNA, which binds to the LS toehold area specifically, HS was replaced, and the middle of the toeholds of LS was exposed for the followed hybridization with FS. Furthermore, the hybridization between FS and LS released the DNA and SS. The released DNA started a new cascading SDR with the other SP. Meanwhile, the released SS formed G-quadruplex structure with NMM and enhanced the fluorescence intensity. With the two-step strand displacement reactions, the method achieved the sensitive detection of HIV-1 DNA with a limit of detection of 1.9 pM. Moreover, it has been successfully applied for the detection of HIV-1 DNA in real biological samples, providing a good application prospect for the early clinical diagnosis of HIV infection. This method also has good reproducibility in human serum with an RSD less than 7% and the recovery rates of different concentrations of HIV-1 DNA from 87 to 103%. In addition, Zhang et al*.* also achieved sensitive detection of HIV DNA through one-step SDR, which used Thioflavin T (ThT) to combine with G-quadruplex structure to obtain the amplified fluorescence signal [31]. The limit of detection for HIV DNA was as low as 13 pM. This method also has a good selectivity and reproducibility, which the RSD value was between 1.3% and 3.5% and the recovery rates from 90 to 99%.

As the strand displacement reaction does not require the presence of enzymes or cofactors, the experimental conditions are relatively simple, and less interference is presented. Therefore, the cyclic signal amplification method based on strand displacement reaction has attracted more and more attention. It usually only needs to design some probes and auxiliary primers to carry out the hybridization reaction, which is economical and cost-effective. However, it also has some disadvantages. In the study of multi-step replacement, multiple hairpin DNA sequences should to be carefully designed, and the optimization of these DNA sequences is tedious to operate. It also needs to consider whether each step in the multi-step reactions can trigger the opening of the hairpin structure, which might cause the failure of the SDR [32–34].

### Enzyme-assisted amplification strategies.

Enzymatic assisted amplification (EAA) refers to the CSA reaction assisted by enzymes for nucleic acids. Many enzymes, such as exonuclease, endonuclease, ligase, polymerase and telomerase, etc., can be used for signal amplification. Different enzymes act in different ways, thus exert different amplification scheme. These enzymes usually utilize probes as the substrate and releases analyte once their enzymatic function is performed. Therefore, the released analyte can trigger another round of activation of probes through the amplification process. With the EAA, the sensitivity and selectivity can be greatly enhanced for fluorescent biosensors [35, 36]. The follow section introduces the major useful enzyme-assisted amplification for fluorescent biosensor, including, exonucleases, endonucleases, CRISPR, polymerase and other.

**Fig. 3:**
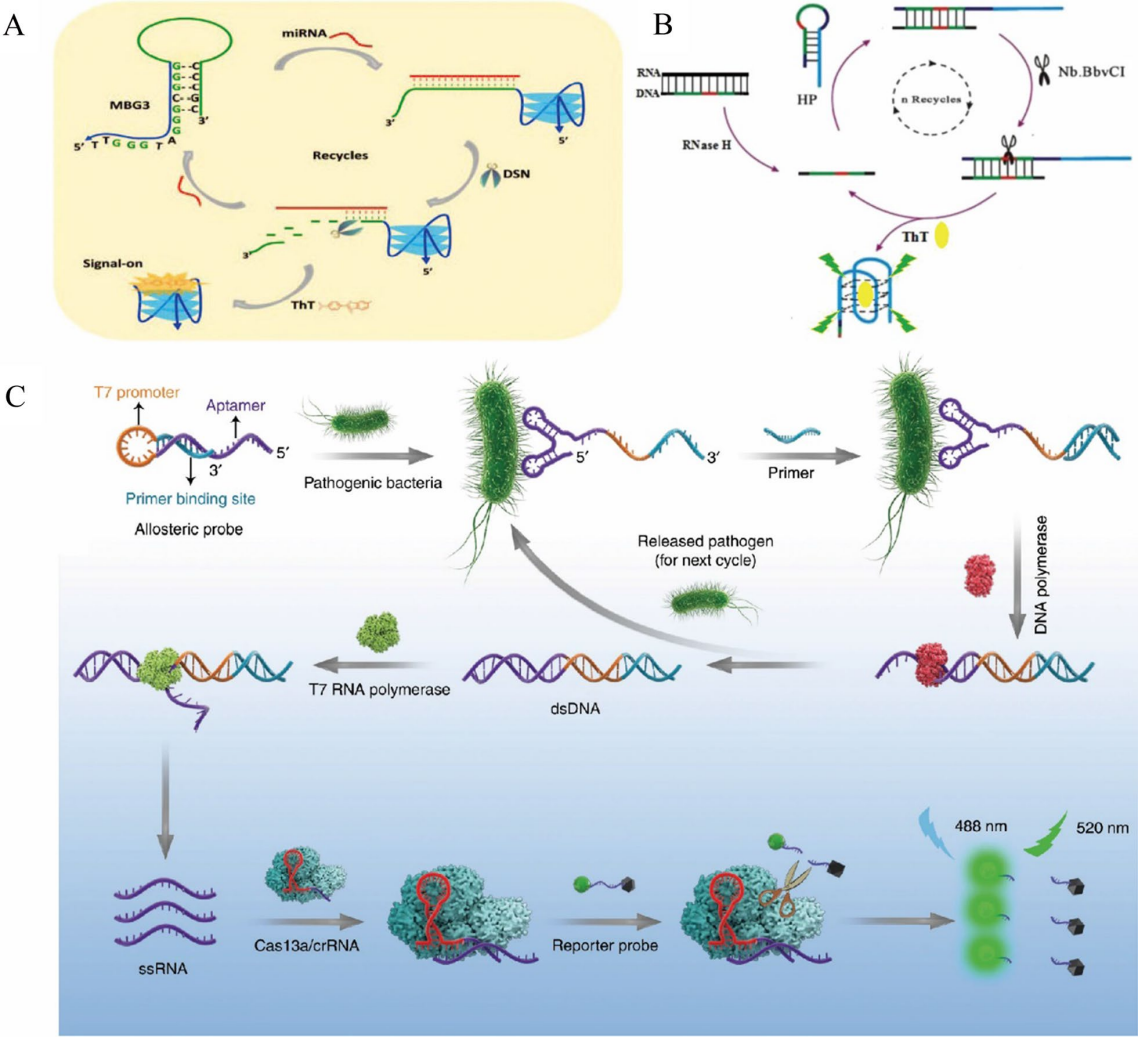
**A** Schematic of sensitivity detection of miRNA by DSN-assisted cyclic amplification technology based on G-triple structure [14]. **B** Schematic diagram of the detection of RNase H by the fluorescence sensor based on EAA [39]. **C** Schematic illustration of sensitive detection of bacterial pathogens using the allosteric probe-initiated catalysis and CRISPR-Cas13a (APC-CAS) system [40]

For example, double-stranded specific nuclease (DSN), which can recognize and digest DNA in double-stranded or DNA-RNA hybrid chains, has no effect on single-stranded DNA, RNA and double-stranded RNA [37]. Zhou et al. proposed a method for sensitive detection of miRNA based on EAA. The detection sensitivity of this method was as low as 1 pM, and it has good applicability to cancer cells samples [38]. Although the EAA improves the detection sensitivity, this method is expensive in probe design. Based on this, Wu et al. proposed a more economical detection method. They designed a molecular beacon based on G-Triplex (MBG3) to achieve sensitive detection of miRNA-141 via DSN assisted circulation [14] (Fig. 3A). The miRNA can hybridize with MBG3 to open its hairpin ring, so the rich G series can form unique triplet structure. Then the DSN can digest MBG3 from the MBG3/miRNA duplex, releasing miRNA and G-triplet. The miRNA can hybridize with another MBG3, and at the same time a large number of G triplets produced can combine with ThT to produce strong fluorescent signal, through the fluorescence signal detection can realize the miRNA sensitive detection, the detection sensitivity was 1 pM, lower than most similar fluorescence detection methods, and the sensor can also be applied to the detection of biomarkers in cell lysates and human urine with the SDR less than 6% and the recovery rates from 90 to 102%.

The nicking enzyme (Nb. BbvCI) is an endonuclease with a specific restriction site 5-GCTGAGG-3. Wu et al*.* achieved the sensitive detection of RNase H by Nb. BbvCI assisted CSA (Fig. 3B) [39]. They designed fully complementary DNA/RNA probes, RNase H can digest the DNA/RNA hybridization double strand, and the released DNA strand can be hybridized with the hairpin probe (HP) to form a specific restriction site, which is recognized and cleaved by Nb.BbvCI. Then G-rich sequences and complete DNA probes were released, ThT fluorescence was enhanced, the DNA probe continued to hybridize with HP into the cycle. The detection limit of this method was 0.03 U mL^−1^ with the recovery rates of different concentrations of RNase H from 94 to 108% in human serums. This method had a potential application prospect in the functional study of RNase H.

In addition, Shen et al*.* implemented sensitive detection of bacterial pathogens using the allosteric probe-initiated catalysis and CRISPR-Cas13a (APC-CAS) system (Fig. 3C) [40]. Allosteric Probe includes T7 promoter, aptamer, and primer binding site. When bacterial pathogens exist, they combined with aptamers and primers hybridized with their binding sites, then dsDNA was produced and the pathogen was released into the next cycle under the action of DNA polymerase. The T7 RNA polymerase then recognized the T7 promoter sequence and initiated transcription to produce large amounts of ssRNA, ssRNA can hybridize with Cas13a /crRNA and activated the collateral cleavage ability of Cas13a /crRNA, thus digesting a large number of reporting probes to enhance the fluorescence signal. This sensor system can sensitively quantify Salmonella Enteritidis cells (from 1 to 10^5^ CFU) in milk, and also distinguish infection in mice by recognizing S. Enteritidis cells in mouse serum. It had potential clinical application value in early diagnosis of pathogens.

There are many kinds of enzymes were applied in the fluorescent sensors based on cyclic signal amplification technology, the enzyme-assisted amplification has lots of advantages, such as the method is simple, economic and effective, without complex instruments, signal amplification effect is well, relatively complete reaction, short time consuming, it also has some shortcomings, such as harsh reaction conditions, different enzyme demand for temperature is different, the cost of the enzyme is high because it is devitalized easily [35, 36, 41, 42].

### Combination of multiple amplification strategies.

The above three cyclic signal amplification methods have their own advantages and disadvantages in biological detection. In order to achieve better signal amplification effect and higher sensitivity for detecting analyte, researchers have gradually developed some methods to combine the two or more CSA methods to detect biomolecules.

**Fig. 4:**
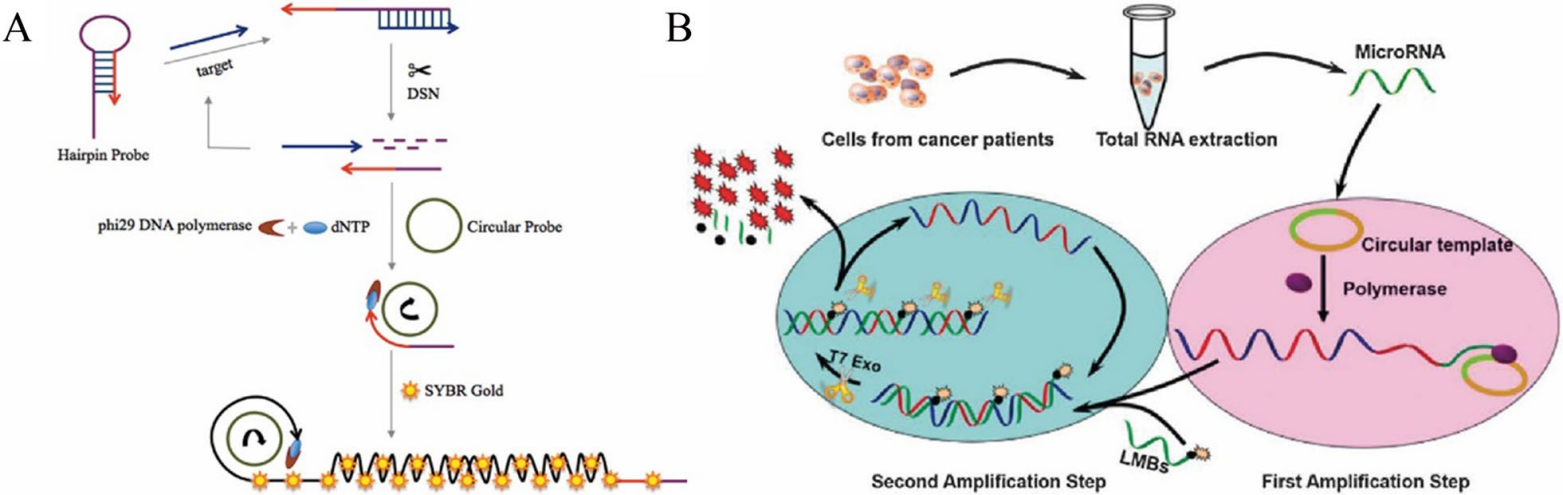
**A** Schematic of DSN-assisted analyte recovery combined with RCA for sensitive detection of circulating miRNA [43]. **B** Schematic of RCA combined with T7 Exo-assisted circulation for sensitive detection of miRNA let-7a [47]

For example, Fan et al. achieved sensitive detection of miRNA by combining DSN assisted circulation with RCA [43] (Fig. 4A). In the presence of the miRNA, hairpin probe (HP) hybridized with the miRNA, and then DSN digested the hybrid complex and released the miRNA, it can realize the cycle process. The remaining HP sequences can be used as primers for the circular probe template, RCA was initiated to produce a large number of long ssDNA strands. They used the SYBR gold dye, which has a lower fluorescence intensity, and combined with ssDNA to enhance the fluorescence signal nearly 1000 times [44–46]. The detection sensitivity of this method was as low as 0.3 fM, the effect was significantly better than one cyclic amplification strategy, and the standard addition recovery rates were from 117 to 122% in human serums. Moreover, this method had a high selectivity for single-base mismatched miRNA, and can also accurately determine circulating miRNA from total circulating miRNA isolated from human serum or tumor cells.

In addition, Cui et al. combined RCA and T7 Exo assisted cyclic amplification to achieve the sensitive detection of miRNA let-7a [47] (Fig. 4B). To reduce background signals and improve hybridization dynamics, linear molecular beacons (LMB) were used to label the fluorophores and quenchants on the terminal and penultimate nucleotides, respectively [48]. In their study, the padlock probes as the circular template were ligated and circularized by the ssDNA ligase. MiRNA, as a primer, initiated the RCA reaction to produce a large number of long ssDNA chains, the long chain can be hybridized with multiple LMBs, then T7 Exo digested LMBs to release fluorophores which maked the fluorescence signal enhanced, the long ssDNA Chains can continue to hybridize with another LMB. The detection limit of this method was as low as 12 fM, and can be applied to the identification of miRNA let-7a expression level in liver cancer cells. What’s more, Xu et al. implemented the sensitive detection of miRNA let-7a by combining RCA and SDR, with the detection sensitivity as low as 76 fM. Detection of the miRNA in total RNA extracted from Hela cells can indicate potential applications in disease detection [49]. In addition, Li et al. achieved the ultra-sensitive and selective determination of miRNA-141 through RCA binding Exo III assisted cycling with taking advantage of graphene oxide (GO)ʹs adsorption and quenching effect, the lower limit of detection estimated to be 100 aM and it showed good selectivity and repeatability in human serum samples with the RSD values of less than 2.7% and the recovery rates from 94.7 to 103.8% [50].

In general, CSA technology can enhance the signal change of the fluorescence sensor several times, the combined application of the two CSA methods can make the fluorescence signal enhancement effect more significant, the detection sensitivity of the analyte is higher than one method, and the deficiency of one CSA method can also be overcome, so as to make more full use of the reaction raw materials. But joint application may involve the design of multiple probes or primers and multiple reaction steps, which will also make the experimental design relatively complex and the experimental cost may increase. Therefore, it is necessary for researchers to reasonably select the cyclic signal amplification technique according to the nature of the analyte and its practical application [43, 49, 50].

## Application of CSA for fluorescent biosensors

The utilization of cyclic signal amplification technology has greatly improved the sensitivity of biosensors and expanded their application fields. Herein, we summarized the recent development of the fluorescence biosensors based on CSA strategies for the detection of important biomolecules, including nucleic acid, protein, enzyme, biological small molecules, metal ions, exosomes and pathogens (Table 2). Some of them are important substances involved in life activities, and some abnormal expression may cause diseases and physiological and pathological changes. All of them can be used as biomarkers, and their sensitive and specific detection is of great significance for clinical diagnosis and treatment of diseases and biomedical research. We also compared the fluorescence sensor based on CSA with other methods such as colorimetry, electrochemical, qPCR, ELASA, non-CSA fluorescence method and so on (Additional file 1: Tables S1–S8). The fluorescence sensors based on CSA are generally faster and more sensitive than other methods.

**Table 2:** Different fluorescent biosensors based on CSA for biomolecules detection

Biomarker	Analyte	Method	Time	Linear range	LOD	Real samples	Refs.
DNA	DNA	EAA (Nicking enzyme)	45 min	0–10^2^ fM	50 fM	Human serum	[58]
	DNA	EAA (RNase H)	1 h	0–50 pM	23 fM	–	[59]
	DNA	SDR	40 min	0–3 pM	0.58 pM	–	[60]
RNA	mRNA	EAA (DSN)	2 h	10^–4^–1 nM	10^2^ fM	Cell lysates	[68]
	miRNA	SDR	2 h	4 × 10^–3^–40 nM	1.48 pM	Cell lysates	[72]
	miRNA	RCA	4.5 h	6.4 7 × 10^4^–10^2^ nM	6.4 pM	Cell lysates and fetal bovine serum	[73]
	lncRNA	EAA (DSN and APE1)	3 h	10^–7^–10^2^ nM	8.1 × 10^–2^ fM	Cell lysates	[78]
	circRNA	RCA	3.5 h	2 × 10^–3^–50 pM	1.1fM	Cell lysates	[84]
	circRNA	EAA (DSN)	30 min	10^–2^–1 pM	10 fM	Cell lysates	[85]
Protein	CEA	EAA (T7 Exo)	65 min	5 × 10^–5^–50 ng mL^−1^	28.5 fg mL^−1^	Human serum	[96]
	AVP	EAA (Nt.AIwI)	30 min	75–7 × 10^2^ pM	75 pM	Fetal bovine serum	[88]
	Insulin	EAA (Exo III)	40 min	4.8 × 10^–2^–2.15 U ml^−1^	4.8 × 10^–2^ U ml^−1^	Fetal bovine serum	[103]
	PSA	EAA (DNAzyme)	2 h	1–100 pg mL^−1^	0.76 pg mL^−1^	Human serum	[104]
Enzyme	Telomerase	EAA (APE1)	3.5 h	1–10^5^ cells	Single cell	Cell lysates	[110]
	Telomerase	EAA (Nt.AlwI)	4.5 h	0–1.5 × 10^–5^ IU	8.93 × 10^–11^ IU	Cell lysates	[111]
	UDG	RCA	> 12 h	5 × 10^–4^– 5 × 10^–2^ U mL^−1^	1.4 × 10^–4^ U mL^−1^	–	[112]
	UDG	SDR	3 h	2 × 10^–4^– 2 × 10^–2^ U mL^−1^	10^–4^ U mL^−1^	–	[114]
	DNA MTase	SDR	4.5 h	10^–5^–1 U mL^−1^	3.3 × 10^–6^ U mL^−1^	Human serum	[115]
	T4 PNK	EAA (Exo III)	5.5 h	5 × 10^–3^–2 × 10^–1^ U mL^−1^	3.3 × 10^–4^ U mL^−1^	Cell lysates and human serum	[35]
Biological small molecules	ATP	SDR and EAA (Nb.BbvCI)	2.5 h	5– 2 × 10^2^ nM	2.2 nM	Human serum	[120]
	ATP	EAA (Exo III)	4 h	20–6 × 10^2^ nM	8.32 nM	–	[121]
	ATP	RCA and EAA (Endo IV)	2.5 h	10^–1^–5 × 10^2^ nM	9 × 10^–2^ nM	Human serum	[122]
	GSH	RCA	3 h	10^2^–10^8^ pM	10 pM	Cell lysates	[126]
	OTA	RCA	> 12 h	5 × 10^–2^–10^2^ ng mL^−1^	10^–2^ ng mL^−1^	Beer samples	[129]
	NF-kB	EAA (Exo III)	1 h	50–10^3^ pM	45.6 pM	Cell lysates	[132]
	Adenosine	EAA (T7 Exo)	1 h	5 × 10^−6^–7 × 10^−4^ mol L^−1^	9.8 × 10^−7^ mol L^−1^	Artificial urine	[134]
Metal ions	Hg^**2+**^	EAA (Exo III)	1.5 h	10^–5^–10^2^ nM	10 fM	River water	[140]
	Hg^**2+**^	SDR	2 h	10^–2^–10 nM	7.9 pM	River water	[141]
	Pb^**2+**^	SDR	30 min	10^−6^–1 mM	0.3 nM	Cell lysates	[144]
	Pb^**2+**^	RCA	1.5 h	1 –10^2^ nM	0.91 nM	Drinking water	[145]
	Cd^**2+**^	SDR	2 h	10^–8^–10^2^ mM	5 pM	Human urine and river water	[148]
	Na^+^	SDR	4 h	10^–1^–12 mM	14 µM	Cell lysates	[136]
Exosomes	Exosome	RCA and EAA (Nb.BbvCI)	3 h	10^3^–10^5^ particles μL^−1^	10^2^ particles μL^−1^	Cell lysates and human serum	[156]
	Exosome	RCA	4.5 h	10^2^–10^6^ particles μL^−1^	42.7 particles μL^−1^	Plasma samples	[157]
	Exosome	EAA (DNase I)	40 min	3 × 10^4^–6 × 10^5^ particles μL^−1^	2.1 × 10^4^ particles μL^−1^	Human serum	[158]
Pathogens	Fn.n	RCA	7 h	7 × 10^–1^ − 7 × 10^4^ ng L^−1^	0.7 ng L^−1^	Fecal specimens	[164]
	S.Typhimurium	SDR	2 h	10–5 × 10^5^ cfu mL^−1^	8 cfu mL^−1^	Milk sample	[166]
	Chlamydia trachomatis	EAA (FEN1)	30 min	0–1 nM	6.7 pM	Human serum	[168]

### Nucleic acid

#### DNA

DNA is the major material basis for storage, replication and transmission of genetic information, it is also an indispensable biological macromolecule for the growth and development of organisms [51–53]. The detection of DNA provide invaluable information for disease diagnosis [54], forensic investigation [55], biomedicine [56], and epigenetics [57]. Therefore, it is very important to develop a rapid, sensitive and efficient detection method for DNA.

**Fig. 5:**
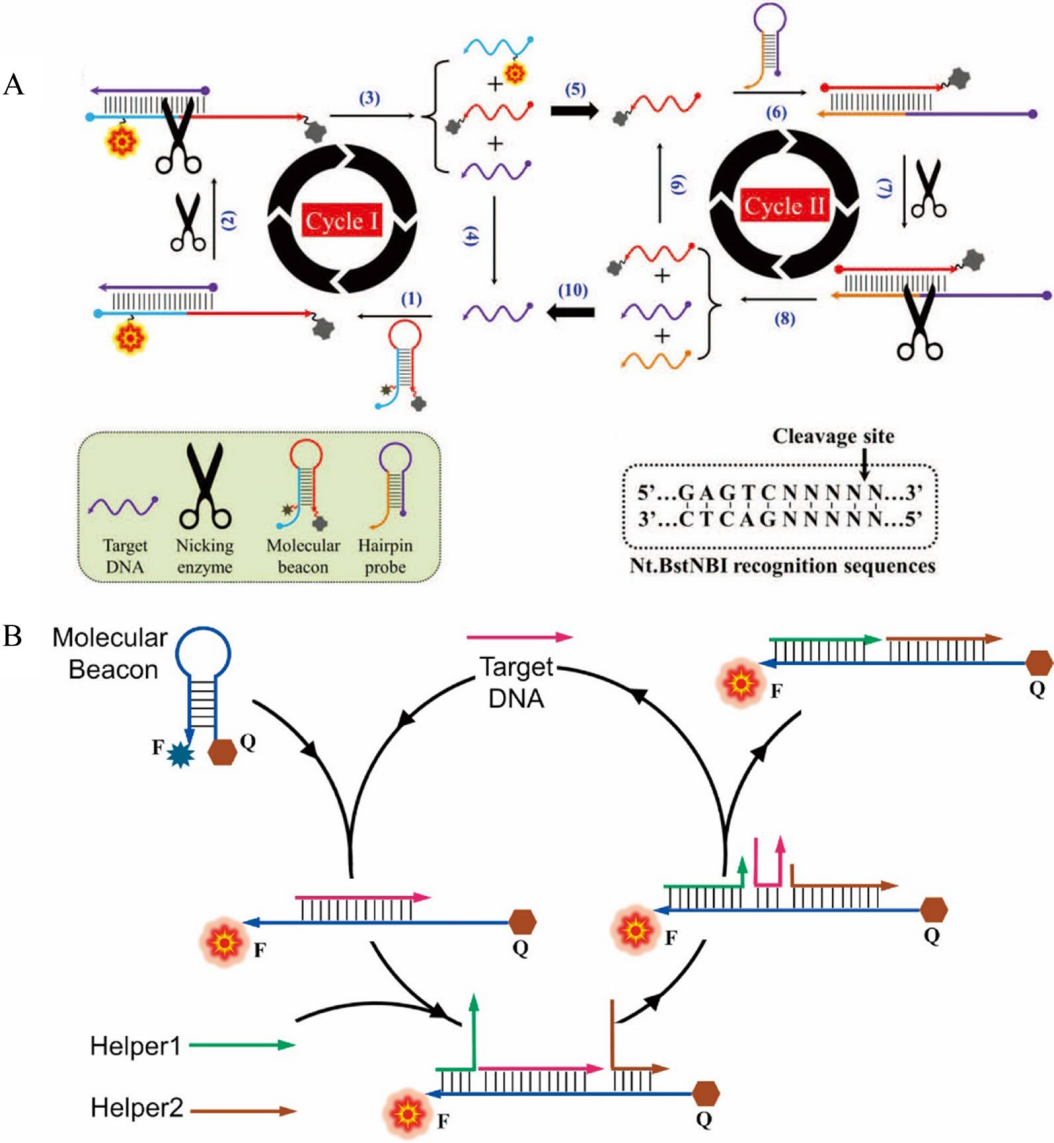
**A** Schematic of Nicking enzyme-assisted biperiodic amplification for DNA sensitivity detection [58]. **B** Schematic diagram of enzyme-free amplified DNA detection based on molecular beacon [60]

For example, Idorenyin A. Iwe et al. constructed a sensitive DNA detection method using the Nicking enzyme assisted double cyclic amplification strategy [58] (Fig. 5A). In their work, the complex formed by the hybridization of the DNA and molecular beacons (MB) became the substrate of the Nicking enzyme, which cleaved the strand of MB. This cleavage not only restored the fluorescence intensity of cleaved MB, but also released the DNA, which can continue to hybridize with MB to realize Cycle I amplification. Moreover, one piece of the cleaved MB hybridized with another hairpin structured probe, which formed the same substrate site of Nicking enzyme. Therefore, the Nicking enzyme digested the hairpin structed probe and generated a piece of DNA sequence identical to the DNA (Cycle II). Therefore, through the Cycle II, more DNA strand with the same sequence of the DNA was produced to trigger the Cycle I again in the system. Through the synergistic amplifications of Cycle I and Cycle II, the sensor achieved the detection of the DNA as low as 50 fM and the recovery tests with fetal bovine serum were from 104 to 111%.

However, the endonuclease requires the certain sequences for the substrates as shown in the previous example. This limits the development of more generous detection methods for DNA detection. Therefore, some nucleases that require no specific sequences for the substrate were constructed for DNA detection. For example, Wang et al*.* designed a fluorescent sensor using fluorescence-labeled RNA MB and RNase H. In this sensor, the RNA MB can hybrid with the DNA, forming the substrate of RNase H. RNase H can only digest the RNA in the DNA/RNA complex, but not the double or single strand RNA or DNA. Therefore, with the help of RNase H, the RNA MB was digested in the presence of the DNA, which released the DNA again to trigger the following digestion of MB. This amplification process based on RNase H significantly enhanced the fluorescence intensity of the probe, make the detection limit as low as 23 fM for the DNA [59], which has been successfully used in the analysis of hemochromatosis gene mutation. Besides the EAA, the enzyme-free amplification was also widely used for the DNA detection. For instance, Huang et al. proposed a method of SDR for DNA detection with higher sensitive than the conventional MB. As shown in Fig. 5B, the sensor composed of a MB and two helper DNA strands. Without the existence of the DNA, the MB was very stable, thus showed quenched fluorescence. However, the addition of the DNA triggered the opening of the MB and then the Helper DNA strands replaced the DNA through the SDR, which released the DNA for the next cycle. Through this SDR amplification, they achieved the detection of the DNA as low as 0.58 pM without the utilization of enzyme and other substrates. Within 30 min reaction, this method demonstrated about 3 orders magnitude better performance than the traditional MB [60]. And we compared CSA-based fluorescence sensors with other methods for DNA detection (Additional file 1: Table S1). The fluorescence sensors based on CSA are generally faster and more sensitive than other methods.

#### RNA

Ribonucleic acid (RNA) is also a genetic information carrier in biological cells and some viruses. RNA involves in transcription and translation to fulfill gene expression [61]. There are many types of RNA, including messenger RNA and non-coding RNA according to different structures and functions. Non-coding RNA can be divided into different subcategories, such as microRNA, lncRNA and circRNA [62]. All of them play different important roles in a variety of cellular processes. The changes of their expression levels are associated with cancer, metabolic diseases, and neurological diseases [63]. Therefore, the development of sensitive detection methods is meaningful for understand the roles of RNA and diagnosis of related disease.

**Fig. 6:**
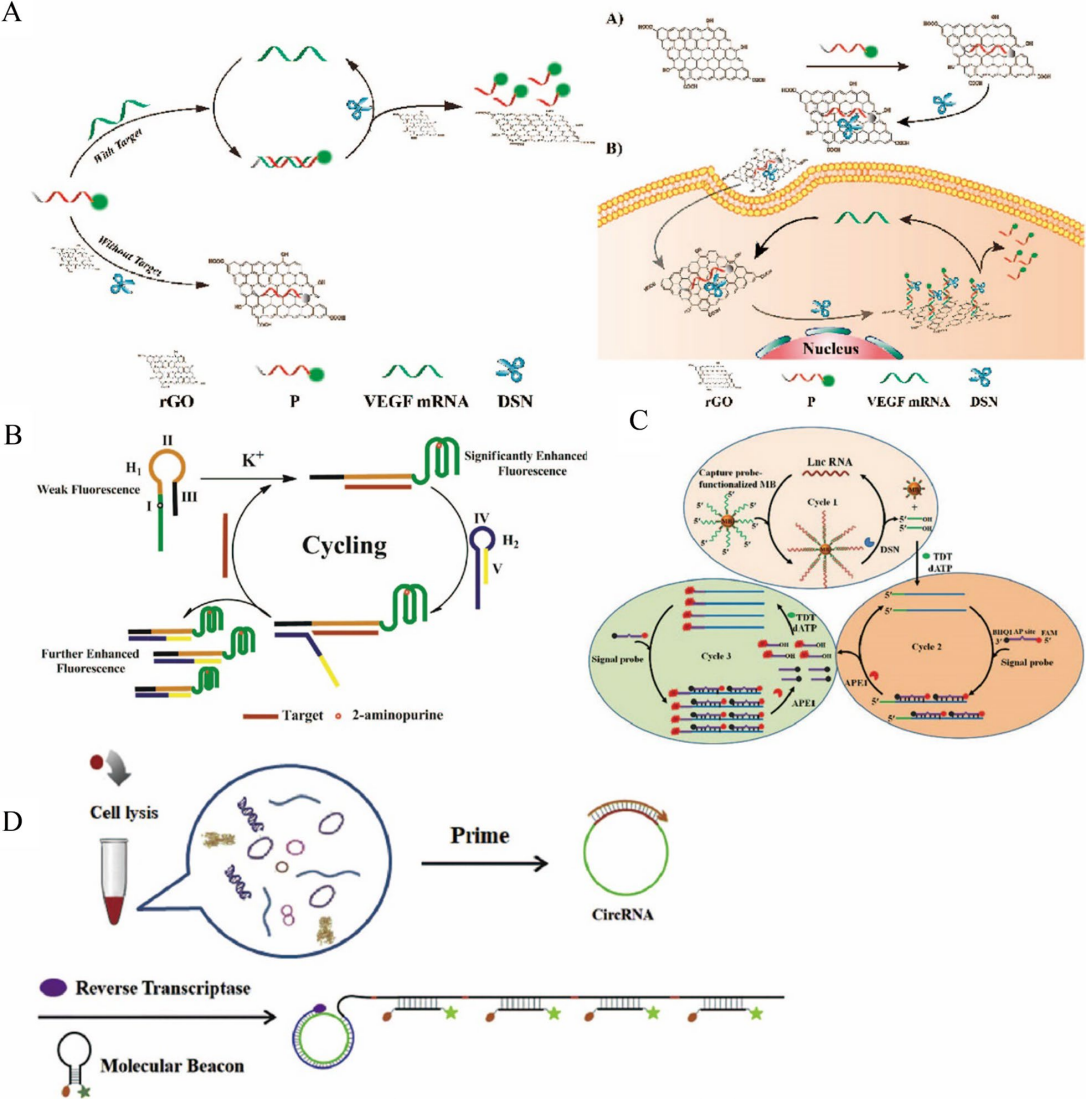
**A** Schematic diagram of sensitive detection of VEGF mRNA and live cell imaging based on DSN-assisted cycling [68]. **B** Schematic diagram of G-quadruple probe based on 2-aminopurine binding SDR for sensitive detection of miRNA-21 [72]. **C** Schematic diagram for sensitive detection of lncRNA based on DSN and APE1 enzyme mediated triple cycle amplification [78]. **D** Schematic diagram for sensitive detection of circRNA based on RCA [84]

Messenger RNA (mRNA) is a direct template for protein biosynthesis. Abnormal expression of mRNA is closely associated with the occurrence and development of various diseases, including cancer [64, 65]. Accurate determination of tumor-related mRNA can monitor the tumor progression and contribute to clinical diagnosis and prognosis [66, 67]. Therefore, the detection of the mRNA is of significance for the disease diagnosis and treatment. For instance, Dang et al*.* used the strong fluorescence quenching ability of reduced graphene oxide (rGO) and DSN-assisted CSA to achieve the sensitive detection of VEGF mRNA in live cells [68] (Fig. 6A). They designed single-stranded DNA probes labeled with FAM that were strongly adsorbed by rGO and thus quenched. When VEGF mRNA was presented, the VEGF mRNA hybridized with the probe, formed the DNA/RNA complex. Therefore, the DNA/RNA complex was released from rGO due to the weak binding force between the double-strand complex and rGO, restoring the fluorescence intensity. Moreover, DSN cleaved the DNA strand of the DNA/RNA hybrid complex and released the VEGF mRNA to trigger the next cycle of hybridization with the probes. With this DSN-based amplification strategy and rGO, the sensor showed high sensitivity for VEGF mRNA with the limit of detection of 100 fM. Due to its excellent biocompatibility and biosafety, the rGO-based sensor was applied for the imaging of VEGF mRNA in live cells. With carefully design of the probe’s sequence, this method can be easily expanded for the detection of other mRNA in cells, which might provide a powerful tool for the disease diagnosis and monitoring.

MicroRNAs (miRNAs) are endogenous non-coding RNAs that are only 18–24 nucleotides in length. Abnormal expression of miRNAs contributes to the progression of viral infection, metabolic diseases and cancer [69]. However, it is difficult to detect miRNAs due to their small molecular weight, low abundance in samples, high similarity in their homologous families, and easy degradation [37, 70, 71]. Therefore, it is of great significance to develop an efficient and sensitive detection method for the study of miRNAs. Li et al*.* proposed a sensitive detection method for miRNA-21 using 2-aminopurine binding G-quadruple probe coupled with SDR [72]. As shown in Fig. 6B, two probes, H1 included the sequence to form the G-quadruple structure with the 2-aminopurine and H2 was used for the displacement reaction. In the presence of the miRNA, H1 hybridized with the miRNA and generated the G-quadruple structure with the assistance of K^+^, which induced the fluorescence enhancement. Then, H2 was open with the released toehold sequence of the H1/miRNA complex, followed by the SDR to release the miRNA. Therefore, the released miRNA triggered another hybridization of H1 to form the second cycle. Through this SDR amplification, numerous of 2-aminopurine-binded G-quadruple structure were formed to generate strong fluorescence intensity. The limit of detection for miRNA by this method was as low as 1.48 pM. Moreover, the sensor was successfully applied for the detection of miRNA-21 in lysates of human breast cancer cells and found that the miRNA-21 was overexpressed in those cancer cells. The detection of miRNA not only can be achieved by the enzyme-free amplification strategies, but also can be constructed with the EAA. For example, Xu et al*.* designed palindromic padlock probes that could be hybridized with miRNAs followed by the RCA process to enhance the fluorescence signal. The combination of RCA products and SYBR Green I produced increased fluorescence intensity without tedious label. Under the optimized condition, the sensor achieved the detection of let-7a miRNA with a limit of detection of 6.4 pM. Furthermore, the sensor was tested to monitor the expression of let-7a miRNA in HeLa cells and showed the similar results with traditional PCR assay. The recovery of the sensor was from 96 to 110%, and the RSD was between 4.8 and 10.6% [73]. And as shown in Additional file 1: Table S2, we compared CSA-based fluorescence sensors with other methods for miRNA detection.

Long non-coding RNA (lncRNA) is a group of endogenous non-protein coding RNAs, whose length is greater than 200 nucleotides. In recent years, many studies have found that lncRNA is involved in many pathophysiological processes and diseases, such as the occurrence and development of malignant tumors [74]. LncRNA, which is located in the nucleus and cytoplasm, can interact with mRNA, miRNA, DNA or protein, etc., and acts as "sponges" to regulate miRNA and prevents its binding to mRNA [75]. LncRNA also regulates the expression of multilevel genes such as post-transcriptional regulation and epigenetic regulation [76, 77]. The abnormal expression of lncRNA has disease and tissue specificity, making lncRNA as biomarkers for disease diagnosis and treatment. Therefore, the development of sensitive detection methods for lncRNA is particularly important. For instance, Zhang et al. proposed a sensitive detection method for lncRNA using DSN and apurinic/apyrimidinic endonuclease (APE1) enzyme-mediated multiple cycles amplification [78] (Fig. 6C). In the Cycle I, they used biotin-labeled DNA probes that bind to streptavidin magnetic beads to capture lncRNA and form RNA/DNA hybrid complexes. DSN can digest the DNA from the complexes to release lncRNA and 3ʹ-OH terminal fragments. LncRNA can hybridize with new Capture DNA probes to release more 3ʹ-OH terminal fragments. Then, in the presence of deoxynucleotidyl transferase (TDT) and dATP, these 3ʹ-OH terminal fragments can form a long polyA sequence, it can be hybridized with a t-rich probe with an AP site, which was cleaved by APE1 to achieve a second cycle and release the fluorophore. The fluorophore with a 3ʹ-OH terminal also can initiate a new TDT-mediated extension reaction in the third cycle. Cycle 2 has realized the continuous enhancement of the fluorescence signal, and Cycle 3 has further enhanced the fluorescence signal, so as to realize the sensitive detection of lncRNA. The detection sensitivity of this method is 0.081 fM, and it can be applied to the detection of endogenous lncRNA, which is helpful for the study of related diseases. This method is more sensitive than the same type of cyclic amplification method, but it involves more cyclic steps and is more complex and time-consuming than the one-step enzymatic cycle reaction experiment.

CircRNA is a non-coding RNA that has attracted much attention in recent years. It participates in the progression of cancer, metabolic diseases and neurological diseases. CircRNA has no 5ʹ terminal end, 3ʹ terminal end and Poly (A) tail, but forms a closed ring through reverse splicing of 3ʹ terminal end and 5ʹ terminal end. Therefore, circRNA is more stable than linear RNA in any environment because it is not easy to be degraded by RNA enzyme [79, 80]. CircRNA can be used as a sponge to adsorb microRNA, and as a bridge to regulate protein–protein interactions, affecting the phosphorylation or ubiquitination of related proteins, or directly translating into polypeptides and participating in epigenetics [81–83]. As an important biomarker for disease diagnosis, it is necessary to develop sensitive detection methods for circRNA detection. Liu et al. proposed a reverse transcription-RCA (RT-RCA) method for sensitively detecting circRNA [84] (Fig. 6D). Using the circRNA as a template, they amplified in the presence of DNA primers and reverse transcriptase to produce a long single strand DNA (ssDNA) containing a large number of repeated sequences. They designed a MB with FAM and Dabcyl marked at the 5ʹ end and the 3ʹ end respectively. One RCA product can be hybridized with multiple MB to open its hairpin structure and enhance the fluorescence signal of the system. The sensitivity of this method is as low as 1.1 fM, which can distinguish circRNA from its linear counterpart, and has been successfully applied to the expression of circRNA in cell lysates, liver cancer tissues, and corresponding para-cancer non-tumor tissues. In addition, Jiao et al*.* proposed a sensitive detection method for circRNA based on DSN assisted cycling [85]. The MB with 6-carboxyuorescein dye (FAM) and Black Hole Quencher 1 (BHQ1) marked at each end can hybridize with circRNA, DSN then digested the hybrid DNA/RNA complex and released circRNA and fluorophore. The limit of detection for circRNA reached 10 fM and it could be applied to cell lysate samples, providing a detection platform for the diagnosis of circRNA related diseases. And we also made use of the CSA technology based on T7 Exo for sensitively detecting circRNA with the limit of detection of 1 pM. The sensor has good reproducibility with the RSD of 3.67% and can apply to cell lysate samples [86]. Taken together, the cyclic signal amplification strategies can be widely used for the sensitive detection of all kinds of nucleic acid.

### Protein

In addition to nucleic acid, proteins are also important components for all cells and tissues. There are many types of proteins with different properties and functions, which are related to many life phenomena [87]. It can provide energy for life activities, maintain metabolism and material transport, regulate the physiological activities, protect organs as antibodies in immune system, etc. [88]. However, abnormal protein expression causes physiological and pathological changes, which is closely related to a variety of diseases [89–91]. Therefore, sensitive detection of proteins as biomarkers is of great significance in medical research and clincial diagnosis. We also compared fluorescence sensors based on CSA with other methods for protein detection, for example, prostate-specific antigen (PSA) (Additional file 1: Table S3).

**Fig. 7:**
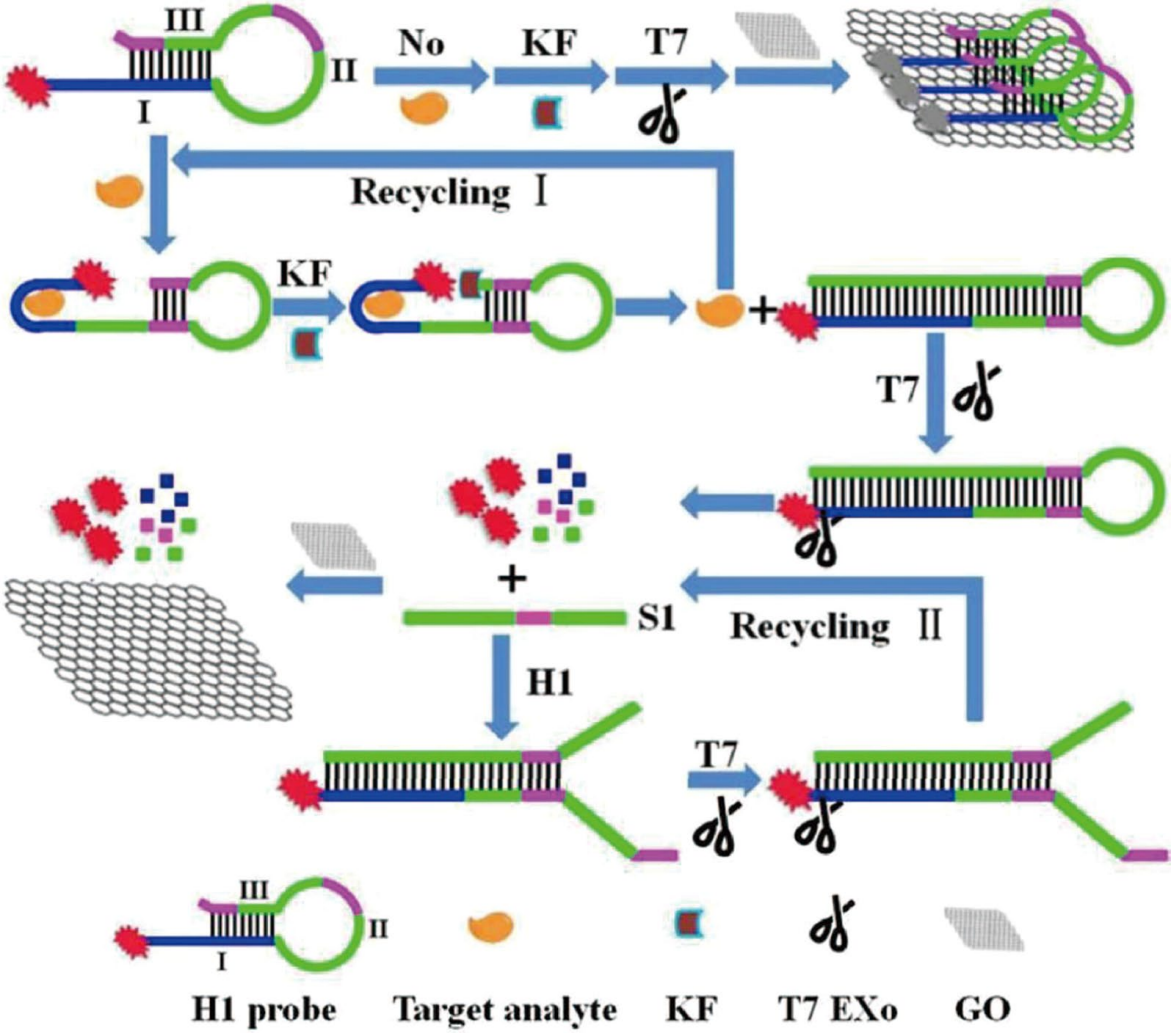
Schematic diagram of CEA sensitive detection by T7 Exo-asssted circulation based on the adsorption quenching effect of GO [96]

Carcinoembryonic antigen (CEA) is an acidic glycoprotein with the characteristics of human embryo antigen, which exists in various body fluids and excreta [92, 93]. The abnormal expression of CEA is related to the possible existence of many types of cancer, which can be used as a biomarker for cancer diagnosis and prognosis [94, 95]. Xu et al. proposed a T7 Exo assisted circulation method based on the adsorption quenching effect of GO to achieve the sensitive detection of CEA with a detection sensitivity of 28 fg mL^−1^ (Fig. 7). The FAM labeled hairpin probe (H1 probe) they designed consists of three parts: sequence of anti-CEA aptamers (I), the loop of the H1 probe (II), part of stem that can be complementary to part I (III), and partly sequences of part II and III are complementary. In the absence of CEA, H1 probe can be adsorbed via π-π stacking interactions by GO. But upon the CEA, H1 probe can bind with CEA to cause conformational changes, then the polymerization took place under the action of Klenow fragment (KF) polymerase, the CEA was displaced to bind with another H1 probe (Cycle I). The polymerization product was digested by T7 Exo to release S1 strands and fluorophore, S1 strands can hybridize with H1 probes and be digested by T7 Exo to release more fluorophore (Cycle II), fluorophore can’t be adsorbed by GO, so that the fluorescence signal of the system is continuously enhanced to realize the sensitive detection of CEA. This method has also been successfully applied to the rapid and direct quantification of serum CEA in colorectal cancer patients and healthy people with the SDR was less than 6.8% and the recovery varied from 95.2 to 106.5% [96].

Arginine-vasopressin (AVP), also known as antidiuretic hormone, regulates intracranial pressure and brain tissue metabolism in the central nervous system, as well as participates in temperature and immune regulation and other physiological functions [97–99]. Tan et al. proposed a sensitive detection method for AVP based on Nt.AIwI nicking enzyme-assisted amplification strategy, with the detection limit as low as 75 pM., and the recovery was 92 ± 2%. This method had great application prospect in monitoring AVP level in biological samples [88]. Insulin, another hormone protein, plays an important role in controlling and regulating blood sugar levels [100]. Abnormal insulin expression is closely related to diabetes. Therefore, accurate measurement of insulin level in blood is of great value for early diagnosis and basic research of hyperglycemia and diabetes [101, 102]. Liu et al. proposed a fluorescence detection system based on exonuclease III double-cycle amplification and graphene oxide (GO) for sensitive detection of insulin [103]. The detection limit of this method is 0.048 U mL^−1^ and it could be used for routine screening of diabetics. Furthermore, a prostate cancer biomarker, prostate specific antigen (PSA) is also a very common target to be detected for cancer screening. For instance, Yan et al. developed a fluorescence sensor based on the Pb^2+^-dependent DNAzyme and GO to realize sensitive detection of PSA, with the detection threshold as low as 0.76 pg mL^−1^ [104]. The recovery of the sensor was obtained in the range of 97.99% to 106.14%, and the RSD was less than 6.39%. In this sensor, the participation of DNAzyme significantly enhanced the detection sensitivity and signal-to-noise ratio through the cyclic amplification process.

### Enzyme

Enzyme is a protein or RNA produced by a living cell that possess highly specific catalytic abilities. Enzymes are biocatalysts that have many functions, such as cell repair, metabolism, immunity improvement, energy generation, etc. [35, 105]. Abnormal expression or weakened activity of enzyme can lead to abnormal reactions, disorders of substance metabolism and diseases [106]. Therefore, it is of great significance to monitor the enzyme’s expression and activity for biomedical applications.

**Fig. 8:**
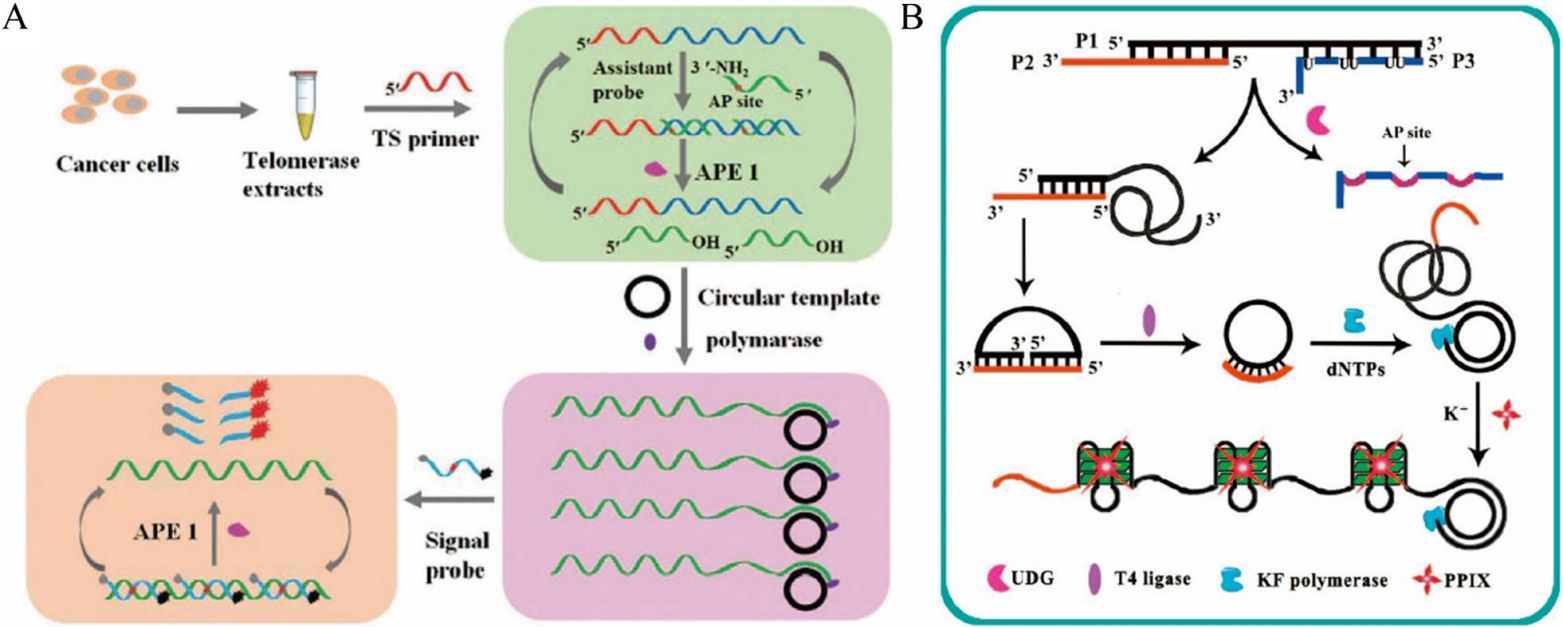
**A** Schematic diagram of sensitive detection of telomerase by enzyme-assisted amplification binding RCA based on telomere triggering [110]. **B** Schematic diagram of sensitively detection of Uracil-DNA glycosylase by RCA [112]

Telomerase plays an important role in maintaining telomere stability, genome integrity, cell activity and proliferation [107, 108]. It has low activity in normal human tissues and cells, but it is activated in tumors and may be involved in malignant transformation. Therefore, it can be used as a biomarker for early diagnosis of cancer [109]. Li et al. proposed a method of multiple signal amplification based on EAA strategy triggered by telomere primers to achieve sensitive detection of telomerase in cancer cells [110] (Fig. 8A). Telomerase recognized and extended the telomerase primers to form long single-stranded DNA, which can hybridize with the probe containing AP sites, then the APE1 can digest the probe from the hybrid complex. The released DNA fragments triggered the RCA process in the presence of the circular template and polymerase, generating a large number of repeated sequences. The signal probe was then hybridized with the RCA products, which were also cleaved by APE1 and continuously restored the fluorescence signal. This method greatly multiplied the fluorescence signal through the three-step cyclic amplification process, realizing the sensitive detection of telomerase at the level of a single cell. Meanwhile, it could be utilized for the screening of telomerase inhibitors. In addition, Liu et al. also achieved sensitive detection of the telomerase by using Nicking enzyme Nt.AlwI assisted triple cyclic amplification. The method possessed a detection limit of 8.93 × 10^–11^ IU, which could be used for both monitor of telomerase activity in cancer cells and telomerase inhibitor selecting [111].

Uracil-DNA glycosylase (UDG) is widely distributed in most organisms and can specifically recognize and remove uracil residues, prevent DNA mutations, and participate in the base removal and repair [105]. It becomes a potential biomarker for the diagnosis of a variety of diseases. Yang et al. implemented the sensitive detection of UDG based on RCA technology [112] (Fig. 8B). The probe they designed contained three parts: a circular DNA template (P1), an RCA primer (P2) and a UDG recognition part (P3). When UDG was presented, UDG specifically identified and removed uracil bases of the P3 to form AP sites and released the P3. Then, the released P1 formed a circular probe in the presence of the T4 DNA ligase and P2, which triggered RCA process with the assistance of the polymerase. As a result, a large number of G-rich sequences were generated to form the G-quadruplex structure under the assistance of K^+^, significantly enhanced the fluorescence intensity of the protoporphyrin IX (PPIX) combined with the G-Quadruplex structure [113]. The sensitivity of this method to detect UDG was as low as 1.4 × 10^–4^ U mL^−1^ and could be used to evaluate the inhibitory effect of uracil glycosylase inhibitors on UDG. In addition, Zhu et al. achieved sensitive detection of UDG based on SDR with a detection lower limit of 10^–4^ U mL^−1^, which was better than most detection methods and could be utilized in the study of UDG function and inhibitor screening. The RSD of this sensor was less than 2.8% [114].

In addition, there are many kinds of enzymes that can be detected sensitively with fluorescent sensors based on CSA technology. Cui et al. presented a sensitive detection method for DNA methyltransferase (MTase) based on the multiple SDR. The detection limit was as low as 3.3 × 10^–6^ U mL^−1^, which was promising for applications in clinical diagnosis. The recoveries of this sensor were observed to be 94% to 111% and the RSD was less than 9.0% in human serum sample [115]. Zhang et al. achieved a sensitive detection of DNA 3ʹ-phosphatase activity using biotin-modified hairpin probes, streptomycin modified magnetic beads, and Exo III-mediated double cycle reactions. A typical DNA 3 ʹ-phosphatase, T4 polynucleotide kinase (T4 PNK) was detected with this method, demonstrating the detection limits of 3.3 × 10^–3^ U mL^−1^. The recovery of T4 PNK were founded between 94 and 110% with the RSD lower than 5.3% in diluted cell extracts. This method offered a promising technology for the detection of DNA 3ʹ -phosphatase activity in complex biological samples [35]. As shown in Additional file 1: Table S4, we compared fluorescence sensors based on CSA with other methods for T4 PNK detection.

### Small biological molecules

Many small biological molecules such as ATP, amino acids, adenosine and vitamins play important roles in human life activities, and their sensitive detection is of great significance in biomedical research and clinical applications.

**Fig. 9:**
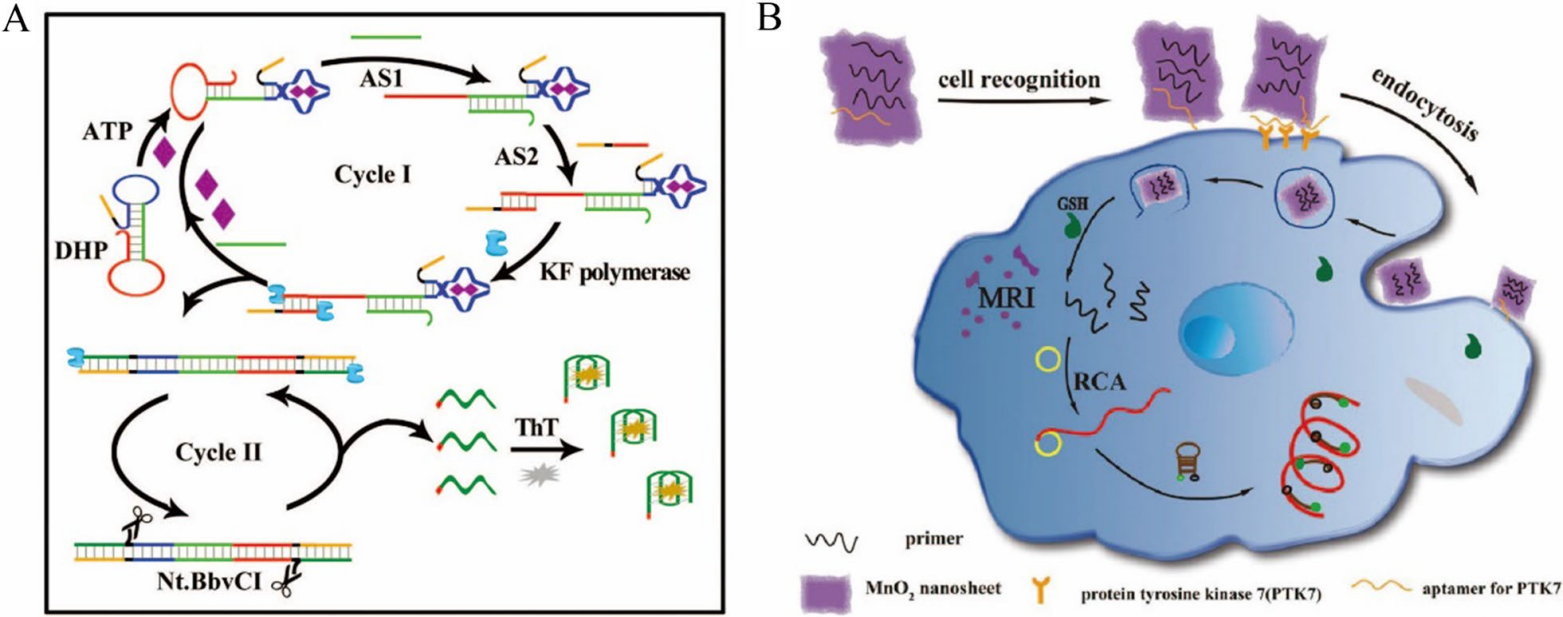
**A** Schematic diagram of Toehold mediated SDR in combination with enzyme-assisted amplification for ATP sensitive detection [120]. **B** Schematic diagram of sensitive detection of GSH and tumor cell imaging using RCA technology based on MnO_2_ nanometer tablets [126]

Adenosine triphosphate (ATP) is a critical small molecule in cells, which is the major energy carrier. It takes part in intracellular energy conversion, biochemical pathways and a variety of biological processes [116]. In clinical settings, abnormal ATP level can be used as a diagnostic indicator of human diseases, such as hypoxia, hypoglycemia, cardiovascular diseases, malignant tumors, etc. [117, 118]. Cell activity and injury can also be evaluated through ATP levels [119]. Therefore, a large number of sensors and kits have been developed for ATP detection, especially in the living cells. For example, Xu et al. proposed a highly sensitive method for the detection of ATP based on the combination of SDR and EAA [120] (Fig. 9A). They designed a double hairpin probe (DHP) to bind with ATP, which exposed the foothold site for Assistance Strand 1 (AS1) through the structure change of the probe. Then, the SDR occurred when AS1 hybridized with toehold complementary sequence to open another hairpin structure. Meanwhile, AS2 hybridized with the complex to form a terminus that favored the action of KF polymerase. During elongation, G-rich sequences were generated and ATP is released into the cycle. In the second cycle, a large number of G-quadruple sequences can be released through the incisor action of Nt.BBvCI and the polymerization of KF polymerase. In the presence of the ThT, the G-quadruplex structures were formed to enhance the fluorescence intensity, which was proportion to the concentration of ATP. This method demonstrated adynamic range of 5–200 nM for ATP, and the limit of detection was 2.2 nM. The recovery of ATP was founded between 98% and 101.8% with the RSD lower than 4.4% in diluted serum samples. Furthermore, this method could be used for the detection of ATP in serum samples. In addition, Ji et al. implemented the sensitive detection of ATP based on EAA. Using a two-step cycle reaction mediated by the DNA polymerase and Exo III, the detection lower limit was 8.32 nM and the RSD was lower than 3.6% [121]. Wang et al. also proposed a sensitive method for detecting ATP based on RCA and Endo IV-assisted signal Amplification. The detection limit of this method was 0.09 nM and utilized it for the detection of ATP in serum samples [122]. Then we compared CSA-based fluorescence sensors with other methods for ATP detection in Additional file 1: Table S5.

Glutathione (GSH) has antioxidant and integrated detoxification effects that help maintain normal immune system function [123]. Changes in its concentration are associated with a number of diseases, including cancer, osteoporosis, Alzheimer's disease, atherosclerosis, and so on [124, 125]. Therefore, the numerous biosensors have been developed for the sensitive detection of GSH, especially in living cells. For instance, Yuan et al. implemented sensitive detection and imaging of GSH in tumor cells based on MnO_2_ nanosheet and RCA technology [126]. As shown in Fig. 9B, MnO_2_ nanosheet adsorbed an aptamer of protein kinase 7 (PTK7) and primer for RCA. When the aptamer binds to PTK7, endocytosis of cells for the probes were promoted, inducing the uptake of the MnO_2_/prime nanosheets into cells. In the presence of the GSH, MnO_2_ nanosheet was degraded into Mn^2+^ that could be used for MRI imaging. Meanwhile, the primers were released to trigger the RCA process with the presence of the Phi29 DNA polymerase, circular DNA template, and dNTPs in cells. As a result, the RCA process produced large quantities of long single-stranded DNA containing repeated sequences, which were hybridized with hairpin DNA probes (5ʹ-end modified FAM and 3ʹ-end modified BHQ1) to produce strong fluorescence signals for the detection and imaging of GSH in cells. The detection limit of this method is as low as 10 pM, which provided a potential application method for intracellular GSH monitoring. However, the introduction of the final DNA probe might cause the unexpected complex of the assay for cellular imaging.

Many other small biological molecules have also been detected with the help of CSA technologies. For example, Ochratoxin A (OTA) is a colorless crystalline compound, a metabolite produced by several aspergillus and penicillium, which is commonly found in cereal crops and related products [127]. OTA is highly toxic, including neurotoxicity, nephrotoxicity, and immunosuppression [128]. Researcher have been utilized the fluorescent technology and CSA technologies to detect OTA for food safety and human health. For example, Hao et al. proposed a sensitive OTA detection method based on RCA technology, which possessed a limit of detection as low as 0.01 ng mL^−1^, and it has been successfully applied to the detection of OTA in beer samples with the recoveries ranged from 94.2% to 105%, providing a potential platform for food safety monitoring [129]. Nuclear factor-kB (NF-kB) is a transcription factor with multiple regulatory roles in gene transcription. It can specifically bind to the K-B sites of many cytokines and gene promoters of adhesion factors or enhancers, then initiate or regulate their transcription. It plays important roles in immune response, inflammatory response, cell growth and development, etc. [130, 131]. Effective detection of its expression level is of great significance for biomedical research and disease diagnosis. In order to detect NF-KB P50, Du et al. applied Exo III assisted circulation to amply the fluorescence signal, achieved the sensitive detection of NF-KB P50 with a limit of detection as low as 45.6 pM. Furthermore, they applied this method for inhibitor screening and cell sample detection [132]. Adenosine is a kind of endogenous nucleoside in human cells. It has physiological effects on cardiovascular system, nervous system and other multiple systems, and is closely related to the occurrence and development of tumors [133]. Wang et al. proposed a sensitive adenosine detection method based on T7 Exo enzyme-assisted amplification, with the lower detection limit of 9.8 × 10^–7^ mol L^−1^, which could be used in artificial urine with the RSD lower than 2.4%, indicating its potential for adenosine detection in clinical diagnosis [134].

### Metal ions

Metal ions are important in our daily life. Some essential metal ions, such as calcium (Ca^2+^), potassium (K^+^), sodium (Na^2+^)and zinc (Zn^2+^), involve in various biological processes [135, 136]. But some metal ions, such as lead (Pb^2+^) and mercury (Hg^2+^), are harmful to living organisms, damaging the nervous system, respiratory system and digestive system. Therefore, it is necessary and significance to develop sensitive methods for metal ions detection. In recent years, more reports have been published on the application of fluorescent sensors in metal ion detection [137].

**Fig. 10:**
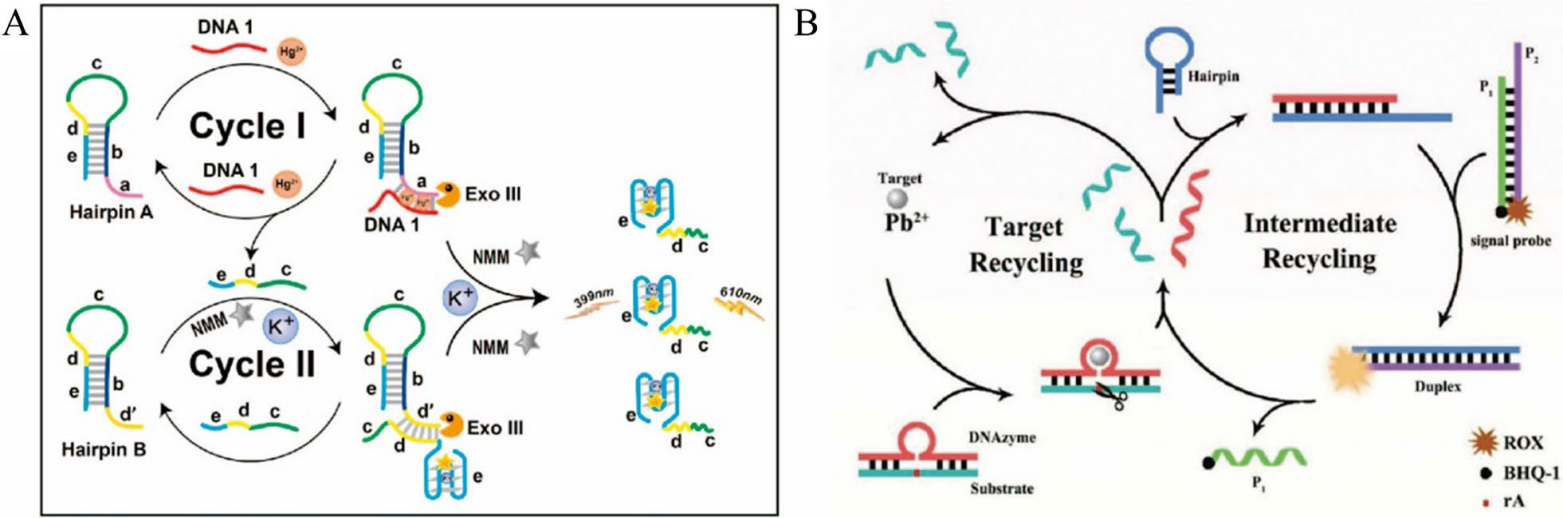
**A** Schematic diagram of Exo III-assisted cycle for sensitively detecting Hg^2+^ [140]. **B** Schematic diagram of SDR for sensitively detecting intracellular Pb^2+^ [144]

Hg^2+^ is considered to be one of the most toxic metal ions to human and is highly permeable to skin, causing severe damage to the central nervous system and respiratory and gastrointestinal problems [10, 138]. Hg^2+^ can specifically combine with thymine (T) residues to form T- Hg^2+^-T complex, which can be used to detect Hg^2+^ [139]. Zhou et al. proposed a sensitive detection method for Hg^2+^ based on Exo -III assisted circulation [140] (Fig. 10A). In the presence of Hg^2+^, DNA1 and Hairpin A (HPA) formed complexes containing T- Hg^2+^ -T, which generated the substrates for Exo III. With the digestion by Exo III, DNA1 was released and fragments containing c, d and e domains from HPA was released as well. Therefore, the released DNA1 could trigger the digestion of another HPA, forming the Cycle I. Meanwhile, the fragments from HPA containing c, d and e domains were hybridized with HPB and then digested by Exo III enzyme as well, forming the Cycle II. Through these two cyclic amplifications, a large number of fragments from HPA and HPB containing c, d and e domains were generated. The free domain E were folded into G-quadruple structure assisted by K^+^ and combined with NMM to produce strong fluorescence signal. With the two-step cycle amplification, sensitivity was significantly improved, the detection limit of this method for Hg^2+^ was 10 fM. Its application in tap water, river water, pond water and other water samples, the RSD and recovery were in the range from 1.2 to 6.4% and 88% to 105%, demonstrated its success and potential for environment monitoring. In addition, Li et al. proposed a sensitive detection method for Hg^2+^ based on SDR with a detection limit of 7.9 pM, which was also used for the detection of actual water samples with the recovery in the range from 94 to 108% and the method was also applicable for the detection of other ions [141].

Lead ion (Pb^2+^) is a major environmental pollutant, which damages circulation system, nervous system and may also cause cancer. Rapid and accurate quantification of lead ions is very important for human health and environment [142, 143]. Wen et al. proposed a sensitive method for intracellular Pb^2+^ detection based on metal ion assisted DNAzyme and SDR [144] (Fig. 10B). In their study, the Pb^2+^ cyclically activated DNAzyme to specifically cleave ribonucleotide (rA) on the substrate. The released fragment of DNAzyme hybridized with the hairpin probes, forming the complex that was then triggered the SDR with the signal probe. Therefore, the quenching group BHQ1 on the signal probe was separated from the fluorophore Carboxy-X-rhodamine (ROX), which enhanced the fluorescence signal of the system. At the same time, the fragment from the DNAzyme was released to trigger the intermediate recycling, amplifying the fluorescence signal again. The detection limit of this method for Pb^2+^ was 0.3 nM. In addition, it was applied to the sensitive detection of lead ion in A-549 and MB-231 cells contaminated with lead, realized the monitoring of Pb^2+^ in living cells. Recently, Tang et al. developed a sensitive detection method for Pb^2+^ based on RCA with a limit of detection as low as 0.91 nM [145]. Also, they applied this method for the detection of Pb^2+^ at different concentrations in drinking water, providing a potential method for the environmental water analysis. As shown in Additional file 1: Table S6, we compared CSA-based fluorescence sensors with other methods for lead ion detection.

In addition to Hg^2+^ and Pb^2+^, cadmium ion (Cd^2+^) is also one of the harmful heavy metals, causing osteoporosis and itai-itai disease even with a very low concentration [146, 147]. Pan et al. designed a multi-step SDR for the sensitive detection of Cd^2+^. With the signal amplification step, the method possessed the limit of detection of 5 pM [148]. Meanwhile, they tested the human urine and river water with different concentrations of Cd ^2+^ using their developed method with the recovery in the range from 95.3 to 107%. Sodium ions (Na^+^) maintain extracellular fluid osmotic pressure, acid–base balance, maintain neuromuscular excitability, and participate in signal transduction and cardiac contraction [149, 150]. Wu et al. also developed a method for the Na^+^ detection based on strand displacement reaction with a detection limit of 14 µM. Meanwhile, they were able to perform intracellular imaging of Na^+^, which could lead to the investigation of the role of metal ions in living organisms [136]. All the above sensors have demonstrated great applications for environmental monitoring and clinical diagnosis related to metal ions.

### Exosome

Exosomes are small vesicles that carry large amounts of proteins, lipids, DNA, RNA, and other biological molecules [151, 152]. Almost all types of cells can secrete exosomes [153]. The function of exosomes depends on the cell type from which they originate. Exosomes are involved in the immune response, cell migration, cell differentiation, angiogenesis, tumor invasion and other aspects [154, 155]. It’s a relatively simple process to extract exosomes from body fluids, so they are of great significance for early diagnosis of diseases. However, their low concentration in the samples also makes early diagnosis difficult, resulting in the development of multi types of detection methods for exosomes. Among them, the fluorescence sensor based on CSA is more concerned because of its simple, fast and sensitive characteristics.

**Fig. 11:**
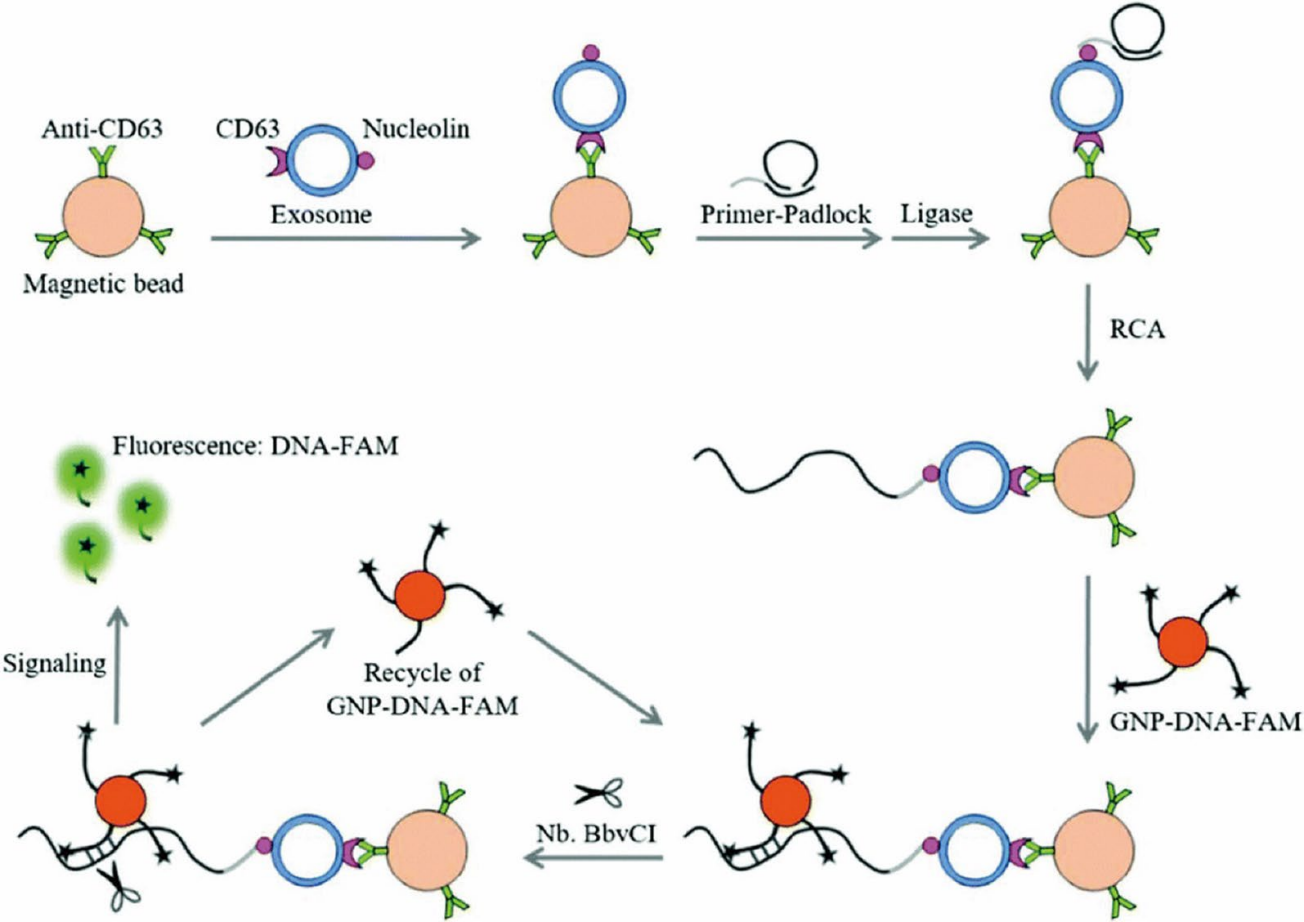
Schematic illustration for the detection of exosomes based on the dual signal amplification by combining RCA and endonuclease-assisted amplification [156]

For example, Huang et al. proposed a method for sensitive detection of exosomes from leukemia sources based on a dual signal amplification strategy, which combined RCA and endonuclease-assisted amplification (Fig. 11) [156]. First, they used an anti-CD63 antibody-modified magnetic bead complex (MB-CD63) to capture exosomes containing CD63 and the nucleolus protein. Then a DNA primer was used as an aptamer to identify "nucleolus proteins" on the exosome surface. With the help of T4 DNA ligase, prime-padlock was ligated and triggered the RCA process, which produced a long DNA sequence with a large number of repeated fragments. The signal probe was constructed by the gold nanoparticles modified with a dye-tagged DNA probe, which was hybridized with RCA products. The complex was recognized and cleaved by Nb·BbvCI, resulting in the release of FAM from gold nanoparticles. Therefore, through the two cycles of amplification, the fluorescence signal in the system was greatly enhanced. The detection limit of this method for exosomes was 100 particles μL^−1^. This method had been successfully applied for the detection of exosomes in serum samples, demonstrating a good clinical application prospect.

In addition, Huang et al. applied RCA for the sensitive detection of exosome originated from gastric cancer cells. They could detect 42.7 particles μL^−1^ in the extraction of plasma from the gastric cancer patients [157]. With the help of graphene oxide (GO), Wang et al. used the DNase I-assisted signal amplification to detect colorectal cancer exosomes, which could detect the cancer-related exosomes as low as 2.1 × 10^4^ particles μL^−1^. The method was validated in serum samples to distinguish between healthy and colon cancer patients [158]. The above methods were also verified in different cancer species, indicating that the fluorescence sensors based on CSA technologies have the potential to be used in the early diagnosis of cancer through the detection of exosomes. The comparison between fluorescence sensors based on CSA with other methods for exosome detection was shown in Additional file 1: Table S7.

### Multiple analytes detection

Detecting multiple analytes simultaneously becomes more important for precise disease diagnosis and prognosis. Therefore, there have been a lot of reports that utilized CSA technologies for multiple analytes detection.

**Fig. 12:**
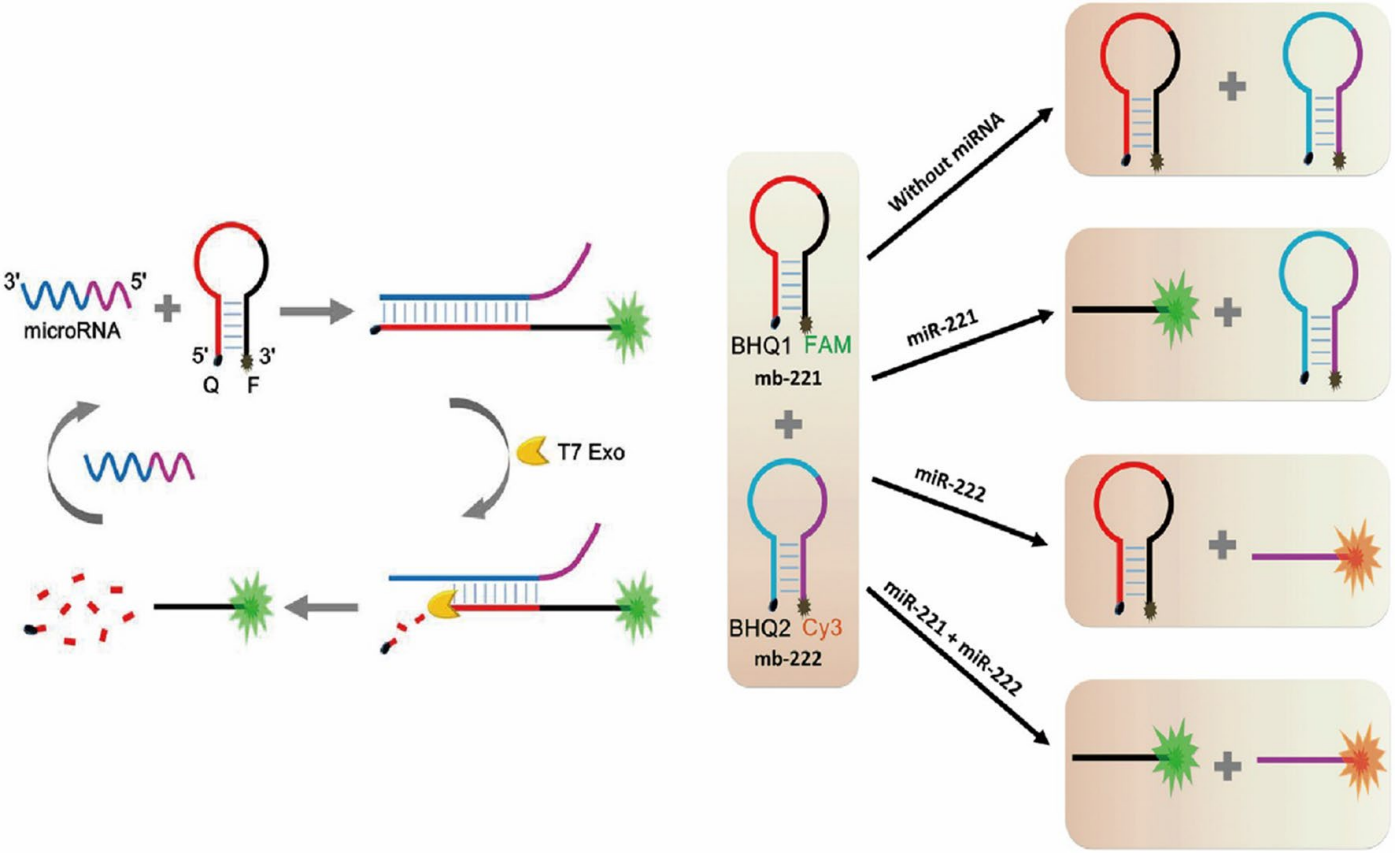
Schematic diagram of simultaneous detection of two different miRNA by T7 Exo assisted circulation fluorescence sensor [16]

For example, Liu et al*.* proposed a method for simultaneous sensitive detection of two microRNAs based on the T7 Exo auxiliary circulation [16]. As shown in Fig. 12, they designed two MB with FAM and Cy3, respectively. In this design, T7 Exo was used as an amplification tool that could only digest the blunt DNA/RNA complex from the 5ʹ terminal. For the intact MBs, T7 Exo had no impact on them. When the miRNA-221 is present, it hybridized with one of the MBs and form the DNA/RNA complex with blunt 5ʹ terminal. After the digestion, the miRNA-221 was released to trigger another round the hybridization and digestion of MB, greatly enhanced the fluorescence intensity. The detection limit for the miRNA-221 by this method was as low as 0.78 pM, which could be used for the detection of the miRNA-221 in HeLa cell. Based on the same principle, the author also designed another MB with the 3ʹ terminal marked with Cy3 to detect miRNA-222. The two fluorescence signals could be used in the reaction system at the same time without affecting each other, which could be applied for multiple analytes detection. This method provides a novel strategy idea for simultaneous detection and clinical diagnosis of multiple miRNAs based on the T7 Exo amplification. In addition, Yao et al. used AuNps-based lateral flow strip and RCA to develop a method for simultaneous detection of miRNA Let 7a and miRNA 21 [159]. The limit of detection was 20 pM and 40 pM respectively. Also, the method was evaluated for the acuate detection of those miRNAs in serum samples.

Besides RNA, viruses could be detected simultaneously with the fluorescent sensors based on CSA. For example, through the RCA process, Du et al. simultaneously detected enterovirus 71 (EV71) and Coxsackievirus B3 (CVB3) with the minimum detection limit of 4.1 × 10^3^ and 1.9 × 10^4^ copies mL^−1^, respectively, which could be used for clinical blood samples [160]. Moreover, Ciftci et al. developed a method to detect three viruses (EBOV, Zika and Dengue) simultaneously by using RCA [161], which showed significant impact on the diagnosis of related diseases.

Compared with the single analyte analysis, multiple analytes detection with the same assay saves time and effort, as well as improve the accuracy of the diagnosis of diseases. However, it also increases the complexity of the design and may cause unexpected hybridization between probes in complex reactions, which may influence the detection results.

### Others

In addition to the detection of biomolecules mentioned above, fluorescent sensors based on CSA can also be applied to the detection of pathogens such as bacteria and chlamydia trachomatis. Additional file 1: Table S8 was showed the comparison between fluorescence sensors based on CSA with other methods for pathogenic bacteria detection. For example, Fusobacterium nucleatum. nucleatum (Fn.n) is widely distributed in the mammalian gut and causes infections in the brain, lungs, mouth, blood or abdomen [162]. It can induce local inflammatory response and increase cytokine expression, leading to the occurrence of colorectal cancer [163]. Therefore, it has been used as a marker for disease diagnosis. In order to detection Fn.n, Jiao et al. proposed a sensitive fluorescent method based on RCA [164]. Through the RCA amplification, the method showed a limit of detection as low as 0.7 ng L^−1^, which was sufficient for the identification of bacteria in real samples. S. Typhimurium, an important zoonotic pathogen, mainly transmits through contaminated food or water [165]. Leng et al. achieved sensitive detection of S. Typhimurium using SDR combined with DNAzyme and silver nanocluster-labeled DNA [166]. Through this method, they could detect S. Typhimurium at the concentration of 8 cfu mL^−1^ and this method showed applicability for S. Typhimurium detection in milk samples, which could be very useful for food safety. What’s more, Chlamydia trachomatis (CT) usually causes trachoma, urogenital tract infections, and sexually transmitted diseases [167]. Yeol et al. proposed a method for the sensitive detection of CT based on flap Endonuclease 1 (FEN1) assisted cyclic amplification combined with GO, and the detection limit of CT was as low as 6.7 pM [168]. Moreover, they applied the method for the detection of CT in human serum samples with the recovery rates in range from 99.4 to 102.4%. Overall, these methods provided some interesting and sensitive testing platforms for food analysis and clinical diagnosis based on cyclic amplification strategies.

## Conclusion and outlook

Fluorescence analysis has attracted more and more attention due to its advantages of simple operation, high sensitivity, high specificity and low cost. The CSA technologies induce stronger fluorescent signal and higher detection sensitivity. Therefore, by taking advantage of CSA technologies, fluorescent sensors have made great progress in biological detection, as well as biomedicine, food safety, environment monitoring, chemistry and other fields. In this review, we have summarized the progress of the fluorescent sensors in the last five years for the detection of different biomolecules through the use of CSA technologies, such as rolling cycle amplification, strain displacement reaction, enzyme-assisted amplification and other CSA technologies. We also compared the fluorescence sensor based on CSA with other methods (Additional file 1: Tables S1–S8).

Although the CSA strategies have made great progress in biological detection, it still faces some problems and challenges. In these assays, some designs are relatively complex and it is hard to sufficiently finish the amplification process, so that the fluorescence signal cannot be enhanced to the maximum level. When actual samples, such as plasma and serum, are tested, the purification steps complicate the whole process. Moreover, although many studies have performed well for detecting the analyte in serum or cell lysate with a relatively ideal RSD and recovery rate, some methods can only achieve semi-quantitative in clinical samples due to the low abundance of the target and the interference of the matrix.

In the future research, there are still some problems that need to be further explored by researchers. For example, the studies involving multiple steps or cycles require more rational design to ensure better utilization of reactive materials. Due to the few studies on simultaneous detection of multiple analytes in a single solution, it is necessary to further develop sensors with different output signals to detect different analytes simultaneously. Some studies have only been verified in buffer, which needs to be more sensitive and specific to avoid interference from other substances in complex samples. Further work is needed to build biocompatible, transferable, and more stable sensors for detection in living cells or in vivo.

In conclusion, we introduced the recent progress of fluorescent sensors for biomolecules based on CSA strategies. Compared with the limitations of traditional biological detection methods, the fluorescent sensors based on CSA technologies provide new ideas for biological detection and benefit clinical diagnosis. The existing challenges need to be continuously studied and solved by researchers in order to achieve greater breakthrough. We believe that with the continuous research and development of fluorescence sensor based on CSA, it will have a broad application prospect and play an important role in practical clinical application in the future.

## Supplementary Information

**Additional file 1.** Comparison of different methods for biomolecules detection.

## Abbreviations

ATP: Adenosine triphosphate
RCA: Rolling circle amplification
SDR: Strand displacement reactions
EAA: Enzyme-assisted amplification
CSA: Cyclic signal amplification
SEF: Surface-enhanced fluorescence
CEA: Carcinoembryonic antigen
FAM: 6-Carboxyuorescein dye
DABCYL: 4-(4-Dimethylaminophenylazo) benzoic acid
Exo I: Exonuclease I
Exo III: Exonuclease III
dNTPs: Deoxynucleotide triphosphate
HS: Hairpin substrate
RSD: Relative standard deviation
ThT: Thioflavin T
FS: Fuel strand (FS)
NMM: *N*-Methylmesoporphyrin IX
SP: Substrate probe
SS: Signal strand
HL: Helper strand
LS: Long strand
DSN: Double-stranded specific nuclease
MBG3: Molecular beacon based on G-Triplex
APC-CAS: Allosteric probe-initiated catalysis and CRISPR-Cas13a
HP: Hairpin probe
GO: Graphene oxide
LMB: Linear molecular beacons
mRNA: Messenger RNA
miRNAs: MicroRNAs
lncRNA: Long non-coding RNA
APE1: Apurinic/apyrimidinic endonuclease
TDT: Deoxynucleotidyl transferase
ssDNA: Single strand DNA
BHQ1: Black Hole Quencher 1
KF: Klenow fragment
AVP: Arginine-vasopressin
PSA: Prostate-specific antigen
UDG: Uracil-DNA glycosylase
PPIX: Protoporphyrin IX
MTase: Methyltransferase
T4 PNK: T4 polynucleotide kinase
AS1: Assistance Strand 1
GSH: Glutathione
PTK7: Protein kinase 7
OTA: Ochratoxin A
NF-kB: Nuclear factor-kB
Ca^2^^+^: Calcium
K^+^: Potassium
Na^2^^+^: Sodium
Zn^2^^+^: Zinc
Pb^2^^+^: Lead
Hg^2^^+^: Mercury
Cd^2+^: Cadmium
ROX: Carboxy-X-rhodamine
EV71: Enterovirus 71
CVB3: Coxsackievirus B3
Fn.n: *Fusobacterium nucleatum*. Nucleatum
CT: Chlamydia trachomatis
FEN1: Flap Endonuclease 1
