# Supplementary material for: Recent advances of fluorescent biosensors based on cyclic signal amplification technology in biomedical detection
Source: J Nanobiotechnology. 2021 Dec 4;19:403. doi: 10.1186/s12951-021-01149-z (PMC8645109; doi:10.1186/s12951-021-01149-z)
Supplement: Supplementary file 1 — Additional file 1. Comparison of different methods for biomolecules detection. [file 12951_2021_1149_MOESM1_ESM.docx]

**Table S1: Comparison of different methods for DNA** **detection.**

| Analytical method | Reaction Time (min) | Linear range (nM) | LOD (nM) | Ref |
| --- | --- | --- | --- | --- |
| DNA Microarrays | > 120 | – | 10^-2^ – 10^-1^ | [1] |
| Colorimetric |  | 10^-2^ – 10^2^ | 10 | [2] |
| Colorimetric | 90 | 2.5 × 10^-3^ – 10^-1^ | 2.5 × 10^-3^ | [3] |
| Electrochemical | 110 | 10^-7^ – 10^2^ | 3 × 10^-8^ | [4] |
| Fluorescence (non-CSA) | 100 | 5 – 2 × 10^2^ | 2.4 | [5] |
| Fluorescence (non-CSA) | 80 | 1 – 5 × 10^2^ | 5 × 10^-1^ | [6] |
| Fluorescence (SDR) | 40 | 10^-1^ – 50 | 1.3 × 10^-2^ | [7] |
| Fluorescence (EAA) | 45 | 0 – 10^-4^ | 5 × 10^-5^ | [8] |

**Table S2: Comparison of different methods for miRNA detection.**

| Analytical method | Reaction Time | Linear range (nM) | LOD | Ref |
| --- | --- | --- | --- | --- |
| Northern blotting | 4 h | – | – | [9] |
| qPCR | 3 h | – | 62 pg μg^-1^ | [10] |
| Colorimetric | 2.5 h | 10^-3^ – 1 | 10^-3^ nM | [11] |
| Electrochemical | 24 h | 10^-4^ – 7 × 10^-2^ | 6 × 10^-5^ nM | [12] |
| Fluorescence (non-CSA) | 3 h | 5 × 10^-1^ – 10 | 1.8 × 10^-1^ nM | [13] |
| Fluorescence (SDR) | 2 h | 4 × 10^-3^ – 40 | 1.48 × 10^-3^ nM | [14] |
| Fluorescence (EAA) | 40 min | 10^-3^ – 5 | 1 × 10^-3^ nM | [15] |
| Fluorescence (EAA and RCA) | 130 min | 0 – 1 | 3 × 10^-7^ nM | [16] |

**Table S3: Comparison of different methods for** **protein detection, for example,** **prostate-specific antigen (PSA).**

| Analytical method | Reaction Time | Linear range (ng mL^-1^) | LOD (ng mL^-1^) | Ref |
| --- | --- | --- | --- | --- |
| Colorimetric | 1 h | 10^-1^ – 10^2^ | 2 × 10^-2^ | [17] |
| Colorimetric | 70 min | 0 – 8 × 10^-1^ | 2 × 10^-2^ | [18] |
| Electrochemical | >12 h | 5 × 10^-2^ – 2 × 10^2^ | 10^-2^ | [19] |
| Microarray immunoassay | 2.5 h | 10^-2^ – 10^2^ | 10^-2^ | [20] |
| Photoelectrochemical | 3.5 h | 10^-2^ – 20 | 3.8 × 10^-3^ | [21] |
| Fluorescence (non-CSA) | 3 h | 10^-2^ – 5 | 3× 10^-3^ | [22] |
| Fluorescence (non-CSA) | 40 min | 5 × 10^-1^ – 3 × 10^2^ | 2 × 10^-1^ | [23] |
| Fluorescence (EAA) | 2 h | 10^-3^ – 10^-1^ | 7.6 × 10^-4^ | [24] |

**Table S4: Comparison of different methods for** **enzyme detection, for example,** **T4 polynucleotide kinase.**

| Analytical method | Reaction Time (h) | Linear range (U mL**^-1^**) | LOD (U mL**^-1^**) | Ref |
| --- | --- | --- | --- | --- |
| Colorimetric | 1.5 | 10^-2^ – 10^2^ | 6 × 10^-2^ | [25] |
| Colorimetric | 1 | 10^-2^ – 8 × 10^-1^ | 1 × 10^-2^ | [26] |
| Electrochemical | >12 | 5 × 10^-2^ – 10 | 2 × 10^-2^ | [27] |
| Electrochemical | 2.5 | 10^-2^ – 1 | 1 × 10^-2^ | [28] |
| Fluorescence (non-CSA) | 1.5 | 5 × 10^-2^ – 10 | 5 × 10^-2^ | [29] |
| Fluorescence (non-CSA) | 4.5 | 0 – 1 | 1 × 10^-2^ | [30] |
| Fluorescence (EAA) | 5.5 | 5 × 10^-3^ – 2 × 10^-1^ | 3.3 × 10^-3^ | [31] |

**Table S5: Comparison of different methods for ATP detection.**

| Analytical method | Reaction Time (h) | Linear range (nM) | LOD (nM) | Ref |
| --- | --- | --- | --- | --- |
| Colorimetric | 2 | 40 – 4 × 10^2^ | 40 | [32] |
| Colorimetric | 2.5 | 10^2^ – 5 × 10^3^ | 61.29 | [33] |
| Electrochemical | 4 | 1 – 2 × 10^2^ | 6 × 10^-1^ | [34] |
| Photoelectrochemical | 1.5 | 10 – 1 × 10^3^ | 3.2 | [35] |
| Fluorescence (non-CSA) | 0.5 | 10^4^ – 3 × 10^6^ | 5 × 10^2^ | [36] |
| Fluorescence (non-CSA) | 2.5 | 0 – 1.4 × 10^6^ | 3 × 10^2^ | [37] |
| Fluorescence (SDR and EAA) | 2.5 | 5 – 2 × 10^2^ | 2.2 | [38] |
| Fluorescence (RCA and EAA) | 1.5 | 10^-1^ – 5 × 10^2^ | 9 × 10^-2^ | [39] |

**Table S6: Comparison of different methods for metal ion detection, for example,** **lead ion.**

| Analytical method | Reaction Time (h) | Linear range (nM) | LOD (nM) | Ref |
| --- | --- | --- | --- | --- |
| Electrochemiluminescence | 1 | 10^2^ – 10^4^ | 70 | [40] |
| Electrochemical | 1 | 5 × 10^2^ – 10^4^ | 3× 10^2^ | [41] |
| Electrochemical | > 12 | 5 -10^2^ | 1 | [42] |
| Ultrasonic assisted precipitation | 5 | 9.7 – 241 | 2.8 | [43] |
| Fluorescence (non-CSA) | 0.5 | 10^2^ – 4 × 10^3^ | 20 | [44] |
| Fluorescence (non-CSA) | 1 | 10 – 10^3^ | 1.043 | [45] |
| Fluorescence (SDR) | 0.5 | 1 – 10^6^ | 3 × 10^-1^ | [46] |
| Fluorescence (RCA) | 1.5 | 1 –10^2^ | 9.1 × 10^-1^ | [47] |

**Table S7: Comparison of different methods for** **exosome detection.**

| Analytical method | Reaction Time | Linear range (particles μL**^-1^)** | LOD (particles μL**^-1^)** | Ref |
| --- | --- | --- | --- | --- |
| Colorimetric | 4.5 h | 5 × 10^3^ – 1 × 10^6^ | 4.46 × 10^3^ | [48] |
| Colorimetric | 1 h | 1.84 × 10^6^ – 2.21 × 10^7^ | 5.2 × 10^5^ | [49] |
| Surface plasmon resonance | 7 h | 10^4^ – 10^11^ | 10^4^ | [50] |
| Electrical | 5 min | 10^7^ – 10^10^ | 10^7^ | [51] |
| Electrochemical | 3.5 h | 7.61 × 10^4^ – 7.61 × 10^8^ | 4.39 × 10^3^ | [52] |
| Fluorescence (non-CSA) | 2 h | 7.5 × 10^4^ – 1.5 × 10^7^ | 4.8 × 10^4^ | [53] |
| Fluorescence (non-CSA) | 5 h | 2 × 10^7^ – 5 × 10^8^ | 2 × 10^7^ | [54] |
| Fluorescence (RCA and EAA) | 3 h | 10^3^ to 10^5^ | 10^2^ | [55] |
| Fluorescence (EAA) | 40 min | 3 × 10^4^ – 6 × 10^5^ | 2.1 × 10**^4^** | [56] |

**Table S8: Comparison of different methods for pathogenic bacteria detection.**

| Analytical method | Reaction Time (h) | Linear range (cfu mL**^-1^)** | LOD (cfu mL**^-1^)** | Ref |
| --- | --- | --- | --- | --- |
| ELISA | 4.5 | 80 – 8 × 10^8^ | 80 | [57] |
| Electrochemical | 4 | 6 × 10^2^ – 6 × 10^6^ | 6 × 10^2^ | [58] |
| Electrochemical | 0.5 | 75 – 7.5 × 10^5^ | 25 | [59] |
| Colorimetric | 3 | 11 – 1.10 × 10^5^ | 11 | [60] |
| RT-PCR | 2.5 | 10^2^–10^6^ | 10^2^ | [61] |
| Multiplex PCR | 12 | 10 – 10^8^ | 1 | [62] |
| Fluorescence (non-CSA) | 5 | 10^5^ – 10^7^ | 10 | [63] |
| Fluorescence (SDR) | 2 | 10 – 5 × 10**^5^** | 8 | [64] |

**References:**

1. Lee HJ, Li Y, Wark AW, Corn RM: Enzymatically amplified surface plasmon resonance imaging detection of DNA by exonuclease III digestion of DNA microarrays. *Anal Chem* 2005, 77:5096-5100.

2. Xiang X, Luo M, Shi L, Ji X, He Z: Droplet-based microscale colorimetric biosensor for multiplexed DNA analysis via a graphene nanoprobe. *Anal Chim Acta* 2012, 751:155-160.

3. Zhou W, Gong X, Xiang Y, Yuan R, Chai Y: Quadratic recycling amplification for label-free and sensitive visual detection of HIV DNA. *Biosens Bioelectron* 2014, 55:220-224.

4. Wang Y, Bai X, Wen W, Zhang X, Wang S: Ultrasensitive Electrochemical Biosensor for HIV Gene Detection Based on Graphene Stabilized Gold Nanoclusters with Exonuclease Amplification. *ACS Appl Mater Interfaces* 2015, 7:18872-18879.

5. Zhang XF, Xu HM, Han L, Li NB, Luo HQ: A Thioflavin T-induced G-Quadruplex Fluorescent Biosensor for Target DNA Detection. *Anal Sci* 2018, 34:149-153.

6. Ge L, Sun X, Hong Q, Li F: Ratiometric NanoCluster Beacon: A Label-Free and Sensitive Fluorescent DNA Detection Platform. *ACS Appl Mater Interfaces* 2017, 9:13102-13110.

7. Zhang F, Xiang L, Xiao X, Chen X, Chen C, Cai C: A rapid label- and enzyme-free G-quadruplex-based fluorescence strategy for highly-sensitive detection of HIV DNA. *Analyst* 2019, 145:206-212.

8. Iwe IA, Li Z, Huang J: A dual-cycling fluorescence scheme for ultrasensitive DNA detection through signal amplification and target regeneration. *Analyst* 2019, 144:2649-2655.

9. Varallyay E, Burgyan J, Havelda Z: MicroRNA detection by northern blotting using locked nucleic acid probes. *Nat Protoc* 2008, 3:190-196.

10. Jin J, Vaud S, Zhelkovsky AM, Posfai J, McReynolds LA: Sensitive and specific miRNA detection method using SplintR Ligase. *Nucleic Acids Res* 2016, 44:e116.

11. Hosseinzadeh E, Ravan H, Mohammadi A, Pourghadamyari H: Colorimetric detection of miRNA-21 by DNAzyme-coupled branched DNA constructs. *Talanta* 2020, 216:120913.

12. Yin H, Zhou Y, Zhang H, Meng X, Ai S: Electrochemical determination of microRNA-21 based on graphene, LNA integrated molecular beacon, AuNPs and biotin multifunctional bio bar codes and enzymatic assay system. *Biosens Bioelectron* 2012, 33:247-253.

13. Zhao J, Jin X, Vdovenko M, Zhang L, Sakharov IY, Zhao S: A WS2 nanosheet based chemiluminescence resonance energy transfer platform for sensing biomolecules. *Chem Commun (Camb)* 2015, 51:11092-11095.

14. Li S, Liu C, Gong H, Chen C, Chen X, Cai C: Simple G-quadruplex-based 2-aminopurine fluorescence probe for highly sensitive and amplified detection of microRNA-21. *Talanta* 2018, 178:974-979.

15. Wu Z, Zhou H, He J, Li M, Ma X, Xue J, Li X, Fan X: G-triplex based molecular beacon with duplex-specific nuclease amplification for the specific detection of microRNA. *Analyst* 2019, 144:5201-5206.

16. Fan T, Mao Y, Liu F, Zhang W, Lin JS, Yin J, Tan Y, Huang X, Jiang Y: Label-free fluorescence detection of circulating microRNAs based on duplex-specific nuclease-assisted target recycling coupled with rolling circle amplification. *Talanta* 2019, 200:480-486.

17. Shayesteh OH, Ghavami R: A novel label-free colorimetric aptasensor for sensitive determination of PSA biomarker using gold nanoparticles and a cationic polymer in human serum. *Spectrochim Acta A Mol Biomol Spectrosc* 2020, 226:117644.

18. Xia N, Deng D, Wang Y, Fang C, Li SJ: Gold nanoparticle-based colorimetric method for the detection of prostate-specific antigen. *Int J Nanomedicine* 2018, 13:2521-2530.

19. Wei B, Mao K, Liu N, Zhang M, Yang Z: Graphene nanocomposites modified electrochemical aptamer sensor for rapid and highly sensitive detection of prostate specific antigen. *Biosens Bioelectron* 2018, 121:41-46.

20. Lee SW, Hosokawa K, Kim S, Laurell T, Maeda M: Simple and robust antibody microarray-based immunoassay platform for sensitive and selective detection of PSA and hK2 toward accurate diagnosis of prostate cancer. *Sensing and Bio-Sensing Research* 2015, 3:105-111.

21. Zhang K, Lv S, Lin Z, Tang D: CdS:Mn quantum dot-functionalized g-C3N4 nanohybrids as signal-generation tags for photoelectrochemical immunoassay of prostate specific antigen coupling DNAzyme concatamer with enzymatic biocatalytic precipitation. *Biosens Bioelectron* 2017, 95:34-40.

22. Zhao Y, Gao W, Ge X, Li S, Du D, Yang H: CdTe@SiO2 signal reporters-based fluorescent immunosensor for quantitative detection of prostate specific antigen. *Anal Chim Acta* 2019, 1057:44-50.

23. Kong RM, Ding L, Wang Z, You J, Qu F: A novel aptamer-functionalized MoS2 nanosheet fluorescent biosensor for sensitive detection of prostate specific antigen. *Anal Bioanal Chem* 2015, 407:369-377.

24. Yan Y, Ma C, Tang Z, Chen M, Zhao H: A novel fluorescent assay based on DNAzyme-assisted detection of prostate specific antigen for signal amplification. *Anal Chim Acta* 2020, 1104:172-179.

25. Jiang C, Yan C, Jiang J, Yu R: Colorimetric assay for T4 polynucleotide kinase activity based on the horseradish peroxidase-mimicking DNAzyme combined with lambda exonuclease cleavage. *Anal Chim Acta* 2013, 766:88-93.

26. Liu H, Ma C, Wang J, Chen H, Wang K: Label-free colorimetric assay for T4 polynucleotide kinase/phosphatase activity and its inhibitors based on G-quadruplex/hemin DNAzyme. *Anal Biochem* 2017, 517:18-21.

27. Hou T, Wang X, Liu X, Pan C, Li F: Sensitive electrochemical assay for T4 polynucleotide kinase activity based on dual-signaling amplification coupled with exonuclease reaction. *Sensors and Actuators B: Chemical* 2014, 202:588-593.

28. Wang Y, He X, Wang K, Ni X, Su J, Chen Z: Ferrocene-functionalized SWCNT for electrochemical detection of T4 polynucleotide kinase activity. *Biosens Bioelectron* 2012, 32:213-218.

29. Lin L, Liu Y, Zhao X, Li J: Sensitive and rapid screening of T4 polynucleotide kinase activity and inhibition based on coupled exonuclease reaction and graphene oxide platform. *Anal Chem* 2011, 83:8396-8402.

30. Gao M, Guo J, Song Y, Zhu Z, Yang CJ: Detection of T4 Polynucleotide Kinase via Allosteric Aptamer Probe Platform. *ACS Appl Mater Interfaces* 2017, 9:38356-38363.

31. Zhang Y, Wang Y, Rizvi SFA, Zhang Y, Zhang Y, Liu X, Zhang H: Detection of DNA 3'-phosphatase activity based on exonuclease III-assisted cascade recycling amplification reaction. *Talanta* 2019, 204:499-506.

32. Zhang L, Guo S, Zhu J, Zhou Z, Li T, Li J, Dong S, Wang E: Engineering DNA Three-Way Junction with Multifunctional Moieties: Sensing Platform for Bioanalysis. *Anal Chem* 2015, 87:11295-11300.

33. Lu S, Wang S, Chen C, Sun J, Yang X: Enzyme-free aptamer/AuNPs-based fluorometric and colorimetric dual-mode detection for ATP. *Sensors and Actuators B: Chemical* 2018, 265:67-74.

34. Li X, Yang J, Xie J, Jiang B, Yuan R, Xiang Y: Cascaded signal amplification via target-triggered formation of aptazyme for sensitive electrochemical detection of ATP. *Biosens Bioelectron* 2018, 102:296-300.

35. Zhang X, Zhao Y, Li S, Zhang S: Photoelectrochemical biosensor for detection of adenosine triphosphate in the extracts of cancer cells. *Chem Commun (Camb)* 2010, 46:9173-9175.

36. Yi M, Yang S, Peng Z, Liu C, Li J, Zhong W, Yang R, Tan W: Two-photon graphene oxide/aptamer nanosensing conjugate for in vitro or in vivo molecular probing. *Anal Chem* 2014, 86:3548-3554.

37. Shen Y, Tian Q, Sun Y, Xu JJ, Ye D, Chen HY: ATP-Activatable Photosensitizer Enables Dual Fluorescence Imaging and Targeted Photodynamic Therapy of Tumor. *Anal Chem* 2017, 89:13610-13617.

38. Xu L, Jiang B, Zhou W, Yuan R, Xiang Y: Coupling strand extension/excision amplification with target recycling enables highly sensitive and aptamer-based label-free sensing of ATP in human serum. *Analyst* 2020, 145:434-439.

39. Wang J, Wang Y, Liu S, Wang H, Zhang X, Song X, Huang J: Duplex featured polymerase-driven concurrent strategy for detecting of ATP based on endonuclease-fueled feedback amplification. *Anal Chim Acta* 2019, 1060:79-87.

40. Dong Y, Tian W, Ren S, Dai R, Chi Y, Chen G: Graphene quantum dots/L-cysteine coreactant electrochemiluminescence system and its application in sensing lead(II) ions. *ACS Appl Mater Interfaces* 2014, 6:1646-1651.

41. Xiao Y, Rowe AA, Plaxco KW: Electrochemical detection of parts-per-billion lead via an electrode-bound DNAzyme assembly. *J Am Chem Soc* 2007, 129:262-263.

42. Shen L, Chen Z, Li Y, He S, Xie S, Xu X, Liang Z, Meng X, Li Q, Zhu Z, et al: Electrochemical DNAzyme sensor for lead based on amplification of DNA-Au bio-bar codes. *Anal Chem* 2008, 80:6323-6328.

43. Abdullah, Balouch A, Talpur FN, Kumar A, Shah MT, Mahar AM, Amina: Synthesis of ultrasonic-assisted lead ion imprinted polymer as a selective sorbent for the removal of Pb2+ in a real water sample. *Microchemical Journal* 2019, 146:1160-1168.

44. Xia J, Lin M, Zuo X, Su S, Wang L, Huang W, Fan C, Huang Q: Metal ion-mediated assembly of DNA nanostructures for cascade fluorescence resonance energy transfer-based fingerprint analysis. *Anal Chem* 2014, 86:7084-7087.

45. Li P, Li J, Bian M, Huo D, Hou C, Yang P, Zhang S, Shen C, Yang M: A redox route for the fluorescence detection of lead ions in sorghum, river water and tap water and a desk study of a paper-based probe. *Analytical Methods* 2018, 10:3256-3262.

46. Wen ZB, Liang WB, Zhuo Y, Xiong CY, Zheng YN, Yuan R, Chai YQ: An efficient target-intermediate recycling amplification strategy for ultrasensitive fluorescence assay of intracellular lead ions. *Chem Commun (Camb)* 2017, 53:7525-7528.

47. Tang D, Xia B, Tang Y, Zhang J, Zhou Q: Metal-ion-induced DNAzyme on magnetic beads for detection of lead(II) by using rolling circle amplification, glucose oxidase, and readout of pH changes. *Microchimica Acta* 2019, 186.

48. Yang Y, Li C, Shi H, Chen T, Wang Z, Li G: A pH-responsive bioassay for paper-based diagnosis of exosomes via mussel-inspired surface chemistry. *Talanta* 2019, 192:325-330.

49. Xia Y, Liu M, Wang L, Yan A, He W, Chen M, Lan J, Xu J, Guan L, Chen J: A visible and colorimetric aptasensor based on DNA-capped single-walled carbon nanotubes for detection of exosomes. *Biosens Bioelectron* 2017, 92:8-15.

50. Zhu S, Li H, Yang M, Pang SW: Highly sensitive detection of exosomes by 3D plasmonic photonic crystal biosensor. *Nanoscale* 2018, 10:19927-19936.

51. Pulikkathodi AK, Sarangadharan I, Lo CY, Chen PH, Chen CC, Wang YL: Miniaturized Biomedical Sensors for Enumeration of Extracellular Vesicles. *Int J Mol Sci* 2018, 19.

52. Xu H, Liao C, Zuo P, Liu Z, Ye BC: Magnetic-Based Microfluidic Device for On-Chip Isolation and Detection of Tumor-Derived Exosomes. *Anal Chem* 2018, 90:13451-13458.

53. He F, Wang J, Yin BC, Ye BC: Quantification of Exosome Based on a Copper-Mediated Signal Amplification Strategy. *Anal Chem* 2018, 90:8072-8079.

54. Zhai LY, Li MX, Pan WL, Chen Y, Li MM, Pang JX, Zheng L, Chen JX, Duan WJ: In Situ Detection of Plasma Exosomal MicroRNA-1246 for Breast Cancer Diagnostics by a Au Nanoflare Probe. *ACS Appl Mater Interfaces* 2018, 10:39478-39486.

55. Huang L, Wang DB, Singh N, Yang F, Gu N, Zhang XE: A dual-signal amplification platform for sensitive fluorescence biosensing of leukemia-derived exosomes. *Nanoscale* 2018, 10:20289-20295.

56. Wang H, Chen H, Huang Z, Li T, Deng A, Kong J: DNase I enzyme-aided fluorescence signal amplification based on graphene oxide-DNA aptamer interactions for colorectal cancer exosome detection. *Talanta* 2018, 184:219-226.

57. Chen R, Huang X, Xu H, Xiong Y, Li Y: Plasmonic Enzyme-Linked Immunosorbent Assay Using Nanospherical Brushes as a Catalase Container for Colorimetric Detection of Ultralow Concentrations of Listeria monocytogenes. *ACS Appl Mater Interfaces* 2015, 7:28632-28639.

58. Wang X, Zhu P, Pi F, Jiang H, Shao J, Zhang Y, Sun X: A Sensitive and simple macrophage-based electrochemical biosensor for evaluating lipopolysaccharide cytotoxicity of pathogenic bacteria. *Biosens Bioelectron* 2016, 81:349-357.

59. Jia F, Duan N, Wu S, Dai R, Wang Z, Li X: Impedimetric Salmonella aptasensor using a glassy carbon electrode modified with an electrodeposited composite consisting of reduced graphene oxide and carbon nanotubes. *Microchimica Acta* 2015, 183:337-344.

60. Wu S, Duan N, Qiu Y, Li J, Wang Z: Colorimetric aptasensor for the detection of Salmonella enterica serovar typhimurium using ZnFe2O4-reduced graphene oxide nanostructures as an effective peroxidase mimetics. *Int J Food Microbiol* 2017, 261:42-48.

61. Miller ND, Draughon FA, D'Souza DH: Real-time reverse-transcriptase--polymerase chain reaction for Salmonella enterica detection from jalapeño and serrano peppers. *Foodborne Pathog Dis* 2010, 7:367-373.

62. Lee N, Kwon KY, Oh SK, Chang HJ, Chun HS, Choi SW: A multiplex PCR assay for simultaneous detection of Escherichia coli O157:H7, Bacillus cereus, Vibrio parahaemolyticus, Salmonella spp., Listeria monocytogenes, and Staphylococcus aureus in Korean ready-to-eat food. *Foodborne Pathog Dis* 2014, 11:574-580.

63. Wen CY, Hu J, Zhang ZL, Tian ZQ, Ou GP, Liao YL, Li Y, Xie M, Sun ZY, Pang DW: One-step sensitive detection of Salmonella typhimurium by coupling magnetic capture and fluorescence identification with functional nanospheres. *Anal Chem* 2013, 85:1223-1230.

64. Leng X, Wang Y, Li R, Liu S, Yao J, Pei Q, Cui X, Tu Y, Tang D, Huang J: Circular exponential amplification of photoinduced electron transfer using hairpin probes, G-quadruplex DNAzyme and silver nanocluster-labeled DNA for ultrasensitive fluorometric determination of pathogenic bacteria. *Microchimica Acta* 2018, 185.
